# Computational Study on Potential Novel Anti-Ebola Virus Protein VP35 Natural Compounds

**DOI:** 10.3390/biomedicines9121796

**Published:** 2021-11-30

**Authors:** Louis K. S. Darko, Emmanuel Broni, Dominic S. Y. Amuzu, Michael D. Wilson, Christian S. Parry, Samuel K. Kwofie

**Affiliations:** 1 Department of Biomedical Engineering, School of Engineering Sciences, College of Basic and Applied Sciences, University of Ghana, PMB LG 77, Legon, Accra LG 77, Ghana or louisdarko20@gmail.com (L.K.S.D.); ebroni002@st.ug.edu.gh (E.B.); 2 Department of Parasitology, Noguchi Memorial Institute for Medical Research (NMIMR), College of Health Sciences (CHS), University of Ghana, P.O. Box LG 581, Legon, Accra LG 581, Ghana; MWilson@noguchi.ug.edu.gh; 3 West African Centre for Cell Biology of Infectious Pathogens, Department of Biochemistry, Cell and Molecular Biology, College of Basic and Applied Sciences, University of Ghana, Accra LG 54, Ghana; dsyamuzu@st.ug.edu.gh; 4 Department of Microbiology, Howard University, Washington, DC 20059, USA; christian.parry@howard.edu

**Keywords:** Ebola virus, molecular docking, molecular dynamics simulations, Ebola virus protein VP35, Ebola virus inhibitors

## Abstract

Ebola virus (EBOV) is one of the most lethal pathogens that can infect humans. The Ebola viral protein VP35 (EBOV VP35) inhibits host IFN-α/β production by interfering with host immune responses to viral invasion and is thus considered as a plausible drug target. The aim of this study was to identify potential novel lead compounds against EBOV VP35 using computational techniques in drug discovery. The 3D structure of the EBOV VP35 with PDB ID: 3FKE was used for molecular docking studies. An integrated library of 7675 African natural product was pre-filtered using ADMET risk, with a threshold of 7 and, as a result, 1470 ligands were obtained for the downstream molecular docking using AutoDock Vina, after an energy minimization of the protein via GROMACS. Five known inhibitors, namely, amodiaquine, chloroquine, gossypetin, taxifolin and EGCG were used as standard control compounds for this study. The area under the curve (AUC) value, evaluating the docking protocol obtained from the receiver operating characteristic (ROC) curve, generated was 0.72, which was considered to be acceptable. The four identified potential lead compounds of NANPDB4048, NANPDB2412, ZINC000095486250 and NANPDB2476 had binding affinities of −8.2, −8.2, −8.1 and −8.0 kcal/mol, respectively, and were predicted to possess desirable antiviral activity including the inhibition of RNA synthesis and membrane permeability, with the probable activity (Pa) being greater than the probable inactivity (Pi) values. The predicted anti-EBOV inhibition efficiency values (IC_50_), found using a random forest classifier, ranged from 3.35 to 11.99 μM, while the Ki values ranged from 0.97 to 1.37 μM. The compounds NANPDB4048 and NANPDB2412 had the lowest binding energy of −8.2 kcal/mol, implying a higher binding affinity to EBOV VP35 which was greater than those of the known inhibitors. The compounds were predicted to possess a low toxicity risk and to possess reasonably good pharmacological profiles. Molecular dynamics (MD) simulations of the protein–ligand complexes, lasting 50 ns, and molecular mechanisms Poisson-Boltzmann surface area (MM-PBSA) calculations corroborated the binding affinities of the identified compounds and identified novel critical interacting residues. The antiviral potential of the molecules could be confirmed experimentally, while the scaffolds could be optimized for the design of future novel anti-EBOV chemotherapeutics.

## 1. Introduction

Ebola virus disease (EVD), formerly Ebola haemorrhagic fever is a rare and severe viral infection with a high mortality rate in humans [1]. EVD was first recorded in 1976 in Zaire, now Democratic Republic of the Congo (DRC) [2]. There was a near simultaneous emergence of the disease in Southern Sudan in 1976. During this time, 284 cases were observed in Sudan and 318 cases were observed in the DRC with a case fatality rate (CFR) of 53% and 88%, respectively [2,3]. Ebola virus, the agent responsible for EVD is named after a village near Ebola river in Zaire/DRC, where the first case was recorded [4,5]. Two different species of the Ebola virus were confirmed, namely EBOV-Zaire (EBOV-Z) and EBOV-Sudan (EBOV-S) [6]. The largest outbreak of EVD so far, from December 2013 to January 2016 resulted in around 28,000 recorded cases, and led to over 11,000 deaths [7]. A re-emergence of the EVD occurred in Gouécké, Nzérékoré Region, Guinea between 18 January and 13 February 2021.

The entry points of Ebola viruses include mucosal surfaces, broken skin, abrasions or by direct parenteral transmission [8]. Laboratory associated and nosocomial infections through needle accidents or exposure to infected materials have also been reported as entry points [9]. The mode of transmission of the virus contributes to the disease outcome, as demonstrated in the 1976 outbreak, for which transmission by injection was 100% as opposed to 80% transmission through contact exposure for the CFR recorded [10]. The potential use of Ebola as a bioweapon with high CFR has led to the extensive study of the pathogenesis of EVD for several years [11,12]. The Ebola virus has been studied in vivo through the use of guinea pigs, rodents and nonhuman primates as well as through in vitro models, providing relevant data to represent infection in humans [13]. Experimentally infected animal models and post mortem studies have showed that upon entry, EBOV infects immune cells such as macrophages, dendritic cells, epithelial cells, and fibroblasts [14]. Prominent characteristics of EVD include viral hemorrhagic fever (VHF), which is characterized by profuse bleeding in infected patients [15,16]. Studies have also shown that the infection of endothelial cells with EBOV does not directly result in hemorrhage, although data on this theory is currently still insufficient [11].

Ebola virus (EBOV) is an enveloped, negative sense, non-segmented, single-stranded, filamentous or thread-like shaped RNA virus which belongs to the Filoviridae family [17]. The genome of the Ebola virus is linearly arranged as follows: 3′-leader-NP-VP35-VP40-GP/sGP-VP30-VP24-Ltrailer- 5′ [18]. The genomic RNA is composed of approximately 19,000 nucleotides, which encode seven structural proteins: glycoprotein (GP), nucleoprotein (NP), RNA polymerase (L), matrix protein (VP40), and three nucleocapsid proteins (VP24, VP30, and VP35) [17,19]. The genomic RNA also encodes two non-structural proteins, the secretory glycoprotein (sGP) and the small secretory glycoprotein (ssGP) [20].

The primary targets of the viral replication are the human immune dendritic cells (DCs) and macrophages, potent antigen presenting cells (APCs), that are found at the site of infection [21]. The viral protein VP35 blocks human type I interferons (IFNs) to prevent the DCs from responding to the viral infection, thereby avoiding the nuclear accumulation of signal transducers, activators of transcription 1 (STAT1), and signal transducers in infected cellular targets. Furthermore, VP35 blocks the activation of the dsRNA-dependent protein kinase receptor (PKR) responsible for the synthesis of IFN [22]. VP35 is reported to successfully block IFN expression by binding to dsRNA via its C-terminal dsRNA-binding domain, which is also referred to as the IFN inhibitory domain [23,24]. The multi-functional protein VP35 is critical for viral replication and virulence [25,26] and therefore, is a plausible drug target. Compounds such as amodiaquine, chloroquine, gossypetin, taxifolin and epigallocatechin gallate (EGCG) have been identified to inhibit the viral replication of the Ebola virus [27,28,29]. Currently, there is only one FDA-approved drug for Ebola virus disease, known as INMAZEB™. It comprises of three monoclonal antibodies, namely, atoltivimab, maftivimab and odesivimab-ebgn [30]. It is thus imperative to identify and validate other therapeutic agents to consolidate and support efforts to facilitate the prevention, management and eradication of EVD. 

Many natural products have been used as therapeutic agents over the past millennium and are currently still in use on a large scale for the treatment of many infectious diseases. Studies have suggested that approximately 50% of new drugs approved by the FDA are either natural compounds or analogs of natural compounds [31]. 

The re-emergence of EVD has led to an accelerated search for potential therapeutics for the treatment of EBOV, which involves the high-throughput screening of hundreds of small molecules within a short period. The African continent possesses a vast and diverse variety of natural flora worthy of exploration to identify natural products that may act as lead compounds in the inhibition of EBOV VP35 [32]. This study aimed to identify potential novel compound leads to inhibit the replication of Ebola virus in host cells through the screening of naturally derived compounds of African origin against the EBOV VP35. In addition, we aim to elucidate novel insights into the mechanisms of binding between the target EBOV VP35 and ligands using molecular dynamic (MD) simulation and MM-PBSA calculations. The biological activity and the mechanisms of action of the potential leads were also predicted to aid in understanding their potential inhibitory roles in viral replication. The drug-likeness of the compounds were further predicted via in silico physicochemical, pharmacological and toxicity profiling.

## 2. Materials and Methods

**Figure 1:**
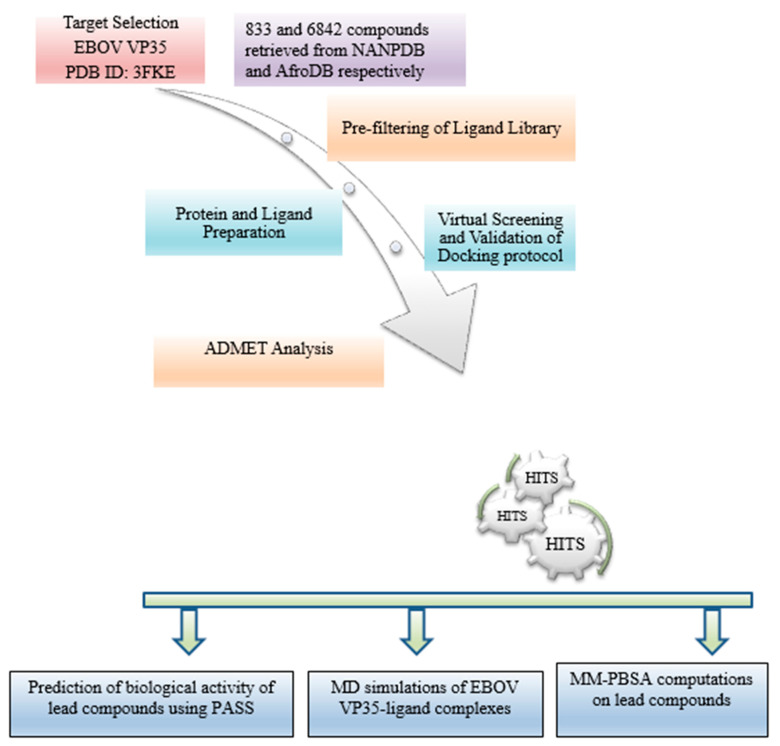
Detailed workflow used to implement this study. The methods involved in this study include molecular docking, analysis of intermolecular interactions, ADMET profiling, prediction of antiviral activity and molecular dynamics simulations.

The schematic workflow used in this study involves the generation of a ligand library, physicochemical and pharmacological profiling, the preparation of the EBOV protein VP35 structure, molecular docking, molecular dynamics (MD) simulations, and predictions on biological activity (Figure 1).

### 2.1. Protein Retrieval

The three-dimensional structure of EBOV VP35, with a resolution of 1.4 Å, was retrieved from the Protein Data Bank (PDB) with the PDB ID: 3FKE [33]. It was visualised and analysed using PyMOL (PyMOL Molecular Graphics System, Version 2.0, Schrödinger, LLC) [34] to remove water molecules.

### 2.2. Retrieval of Compounds from Natural Products Databases

A total of 7675 compounds were retrieved from the library of African Medicinal Plants (AfroDB) and Northern African Natural Products Database (NANPDB) [35,36]. The library consisted of 6842 compounds that were obtained from NANPDB, and 833 compounds from the ZINC database catalogue of AfroDB. The natural compounds were labeled with the prefixes ‘ZINC’ or ‘NANPDB’ to represent their respective libraries. Amodiaquine, chloroquine, gossypetin, taxifolin and epigallocatechin gallate (EGCG) were identified as known compounds that could inhibit the viral replication of the Ebola virus via EBOV VP35 [27,28,29]. 

### 2.3. Protein Active Site Evaluation

The active site of the EBOV VP35 was characterised through the Computed Atlas of Surface Topography of proteins (CASTp) [37] (available online: http://sts.bioe.uic.edu/castp/calculation.html accessed on 20 March, 2020), and analysed with Chimera version 1.12 [38]. The binding pocket of the EBOV VP35 is characterised by surface area, volume and the cavities in a solvent [39].

### 2.4. Pre-Filtering of Ligand Library

Ligands were filtered using ADMET Predictor version 8.0 [40] to ensure compliance with the Lipinski’s Rule of five (RO5), which is as follows: compounds with a molecular mass of less than 500 Da, have no more than 5 hydrogen bond donors, no more than 10 hydrogen bond acceptors, and the octanol-water partition coefficient log is no greater than 5 [41,42]. The ADMET Risk model helps to identify any potential liabilities that are likely to impede the success of the prospective drug design. The ADMET risk ranges from 0 to 24, whereby scores of less than 7 indicate the characteristics of the compound to be more drug-like [43].

### 2.5. Protein and Ligand Preparation

The energy minimisation of the protein was performed using the GROningen MAchine for Chemical Simulations (GROMACS version 2018), and by utilising Optimized Potentials for Liquid Simulations (OPLS)/All Atom (AA) force field [19,44]. The GROMACS file, in a ‘gro’ format, of the minimised protein was visualised in PyMOL and the structure was exported in accordance with the Protein Data Bank format (‘.pdb’). The ligands were retrieved in a structure data file ‘.sdf’ format and imported to the Open Babel module in PyRx, where they were subject to energy minimisation over 200 steps using an MMFF94 force field and Conjugate gradient algorithm. The ligands and EBOV VP35 protein were finally converted into the Protein Data Bank partial charge and atom type ‘.pdbqt’ file format for docking [45].

### 2.6. Virtual Screening and Validation of Docking Protocol

The Auto-Dock Vina module, embedded in PyRx [46], was used to screen the compounds against the EBOV VP35 protein and the complexes were analysed using PyMOL [34,47]. The grid box was maximised to cover all the binding sites of the protein, with the following dimensions and spacing: center_x = 48.7160 Å, center_y = 48.6696 Å and center_z = 48.7246 Å, and size_x = 139 Å, size_y = 95 Å and size_z = 190 Å. The ability to discriminate between active compounds and decoys was essential in evaluating the performance of the docking protocol [48]. As such, five known EBOV VP35 inhibitors including amodiaquine, chloroquine, EGCG, gossypetin and taxifolin were used to generate 50 decoys, each from the Directory of useful decoys, and enhanced (DUDE) (available: http://dude.docking.org accessed on 9 April 2020) [49]. The decoys possess different 2D topologies but similar physiochemical properties. The area under the curve (AUC) for the Receiver Operator Characteristic (ROC) curve was generated using easyROC (version 1.3) [50] after screening 250 decoys and 5 known inhibitors against the EBOV VP35 protein. This was performed to evaluate the performance of the docking tool [49,51].

### 2.7. Molecular Interaction Profiling 

The Protein–ligand interactions of the compounds and EBOV VP35 were predicted using Discovery Studio (DS) and Maestro in Schrödinger suite [52,53]. The distance between the interacting amino acid residues of the protein and the ligand atoms were then calculated. A 2D schematic diagram was generated to represent the hydrogen and hydrophobic interactions. 

### 2.8. Pharmacokinetic Profiling

In order to assess their pharmacokinetic properties and drug-likeness, the hit compounds were screened using SwissADME as described previously [54]. The pharmacokinetic properties such as gastro intestinal (GI) absorption, the crossing of the blood–brain barrier (BBB), p-glycoprotein and the inhibition of isoforms of the cytochrome P450 (CYP) family were analyzed. In order to determine the drug-likeness of the compounds, parameters such as RO5, Veber’s and Ghose rules were applied [55,56]. Promising compounds and five known inhibitors were selected for toxicity evaluations that were conducted using OSIRIS Property Explorer in DataWarrior version 4.7.2, to determine their mutagenic, tumorigenic, reproductive and irritant traits [57]. 

### 2.9. Prediction of Anti-Viral Activity of Lead Compounds

The Prediction of Activity Spectra for Substances (PASS) tool was used to characterize the biological activity of the compounds using their structures in the SMILES file format [58,59]. The anti-EBOV inhibition efficiency was predicted using the SDF files of the compounds via a random forest-based model [60].

### 2.10. Quality and Efficiency of Evaluation of Potential Lead Compounds

Their compound-level efficiency metrics were computed to determine the quality of the selected compounds [61]. Additionally, the ligand efficiency (LE) is used to measure the effectiveness of the compounds relative to the size of the protein. This is expressed in Equations (1)–(5):

(1) $$\mathrm{Ligand}\;\mathrm{Efficiency}\;\left(\mathrm{LE}\right)=\frac{\Delta G}{\mathrm{HA}},$$

∆G is the binding energy of the compound [61] and HA is the number of heavy atoms.

Additionally, specific ligand quality indices such as Fit Quality (FQ), the inhibitory constant (Ki), ligand efficiency-dependent lipophilicity (LELP) and the ligand efficiency scale were also calculated [62,63].

(2) $$\mathrm{Fit}\;\mathrm{Quality}\;\left(\mathrm{FQ}\right)=\frac{\mathrm{LE}}{\mathrm{LE}\;\mathrm{Scale}},$$

(3) $$\mathrm{Ligand}\;\mathrm{Efficiency}\;\mathrm{Scale}=0873\times e^{-0.026\times \mathrm{HA}}-0.06,$$

(4) $$\mathrm{Ligand}\;\mathrm{Efficiency}\;\mathrm{Dependent}\;\mathrm{Lipophilicity}\;\left(\mathrm{LELP}\right)=\frac{\log P}{\mathrm{LE}},$$

(5) $$\mathrm{Ki}\;={\;e}^{\frac{-\Delta G}{\mathrm{RT}}},$$

R is a gas constant of 1.987 × 10^−3^ kcal/K-mol; T represents the absolute temperature of 298.15 K [64], and Ki denotes the inhibitory constant.

### 2.11. Molecular Dynamic Simulations of Protein-Ligand Complexes

The top four ligand–protein and two known inhibitor-protein complexes underwent MD using GROMACS version 2018 [19]. The protein complexes were simulated for a 50 ns timescale to provide insight into their modes of interaction and the ways in which they behave dynamically [65]. The six complexes were prepared using a CHARMM36 all-atom force field. The charge topology of the compounds was generated using the CGenFF [66] and solvated in a cubic boundary box with a distance of 1.0 nm, using the TIP3P water model [67]. The charged system was neutralized with the precise addition of concentrations of chloride (Cl^−^) ions and its energy was minimized at 10 kJ/mol/nm using the steepest descent algorithm for 1000 steps to prevent steric clashes [68]. Furthermore, the systems were subjected to equilibration by position-restrained dynamics simulations (via NVT and NPT ensemble) at a constant temperature of 300K and a pressure of 1 atm for 1000 ps [69]. Finally, MD simulation was conducted for all the complexes, for 50 ns, with time steps of 2 fs. The radius of gyration (Rg) and the root mean square deviation (RMSD) graphs were plotted using XMGRACE [70,71]. 

### 2.12. Binding Free Energy Calculations of Protein-Ligand Complexes by MM-PBSA

The MM-PBSA approach was used to calculate the binding free energies of the complexes, employing both the continuum solvent models and the molecular mechanics extracted from MD simulations. MM-PBSA calculations were carried out using GMXPBSA [72] script and were statistically analyzed using the R programming package [73].

## 3. Results

### 3.1. Structural and Binding Site Analysis

**Figure 2:**
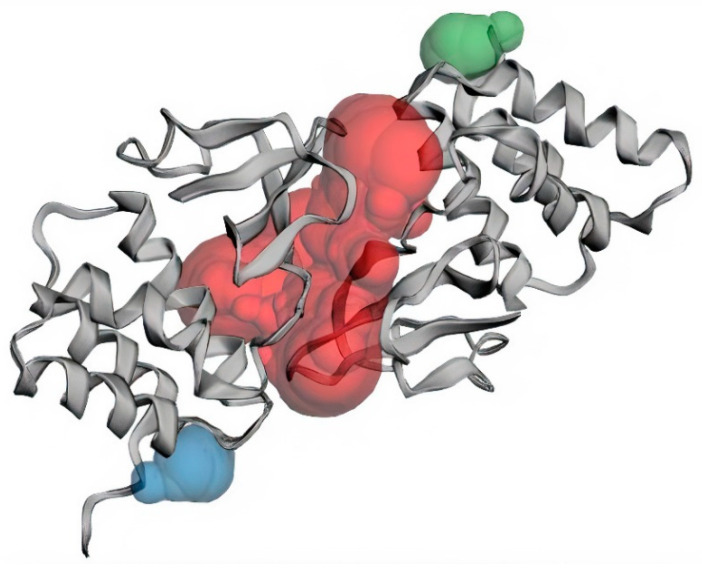
Three major binding pockets of EBOV VP35 predicted via CASTp, with large surface areas and volumes. The red, blue and green colors represent pockets 1, 2 and 3, respectively.

The structure of EBOV VP35 C-terminal domain (also known as the interferon inhibitory domain, IID), with a high resolution of 1.40 Å, was obtained from the PDB database with ID 3FKE [74]. The structure is comprised of 2 subdomains which are an N-proximal α-helical subdomain and a C-terminal β-sheet subdomain, of approximately 120 residues each [74]. The residues lining the binding sites of EBOV VP35 were predicted using CASTp (Table 1). Three major binding pockets were also predicted via CASTp (Figure 2 and Table 1), which were then analyzed using Chimera version 1.12. Pockets with a small surface area and volume were not considered as reasonable binding pockets for the virtual screening of ligands. Pocket 2 and 3 are located on chains B and A of the protein, respectively, while pocket 1 is composed of residues found on both chains (Table 1). Previous structural studies identified residues lining the carboxy-terminal dsRNA-binding domain of EBOV VP35 as critical for the viral polymerase cofactor function [75,76,77,78]. Residues Lys319, Arg322, and Lys339 of the EBOV VP35 have previously been identified as critical residues for dsRNA binding [33,74]. 

The EBOV VP35 structure has two basic patches, the first basic patch (FBP) and the central basic patch (CBP), which are highly conserved among Ebola virus species. The FBP is crucial for molecular interactions with the Ebola virus nucleoprotein and VP35 polymerase cofactor function, whilst the CBP is involved in dsRNA binding and the inhibition of IFN [79]. Residues Ala221, Arg225, Gln241, Leu242, Lys248, Lys251, Pro293, Ile295, Ile297, Asp302 and Phe328 are located near and inside the FBP groove [79,80]. A recent study also identified Ala221, Arg225, Gln241, Leu242, Lys248, Lys251, Pro293, Pro292, Ile295, Ile297, His296, Asp302, Phe328 Ala238, Val245, Ile246, Leu249, Ile278, Ile280, Phe287, Ala306, Cys307, Pro315, Pro318, Ile320, Asp321, Gly323, Trp324, Val325, Leu338 and Ile340 as residues that line the binding site of the VP35 protein, based on their literature review and by visualizing the protein structure [81]. These residues are consistent with pocket 1 as predicted via CASTp (Table 1).

### 3.2. Molecular Docking Studies

A total of 1470 pre-filtered ligands and five known EBOV VP35 inhibitors comprising amodiaquine, chloroquine, gossypetin, taxifolin and epigallocatechin gallate (EGCG) were docked in the binding pockets of the EBOV VP35 protein. The docking protocol was validated using a ROC curve, which was computed after the screening of decoys and obtained using DUDE, against the EBOV VP35. The area under the curve (AUC) represents the ability of the docking tool to distinguish between active ligands and decoys [82]. AUC values range between 0 and 1; where values closer to 1 indicate a higher discrimination potential and 0 represents poor discrimination [83]. The docking protocol was validated with an AUC of 0.72 (Figure S1), thereby indicating the high discrimination potential of AutoDock Vina to distinguish between active and inactive compounds.

Although a recent study employed a barrier of −7.0 kcal/mol to shortlist compounds, a more stringent threshold of −8.0 kcal/mol was used in this study [84] to enhance the classification between specific and non-specific binding [85]. Ninety-four compounds with binding energies of −8.0 kcal/mol or below were selected for further analysis. These binding energies indicate the high binding affinity of the compounds to EBOV VP35. The ability of a compound to successfully bind is an indication of its potential inhibition [86]. All the compounds shortlisted had a greater binding affinity than that of the known inhibitors except for EGCG, which had a binding affinity of −8.1 kcal/mol (Table 2). Amodiaquine and chloroquine are FDA-approved drugs that are found to be inhibitors of the viral replication of EBOV while the anti-EBOV compounds EGCG, gossypetin and taxifolin have yet to be approved [27,28,29]. Additionally, these compounds possessed better binding affinities in this study than in previous studies [80,81] (Table 2).

### 3.3. ADMET Profiling for Identification of Drug-Likeliness

The compounds were physicochemically profiled using Swiss ADME and OSIRIS Property Explorer. RO5, Ghose and Veber’s rules were used to evaluate the drug-likeness of the compounds [87]. Most of the compounds that violated more than one drug-like parameter were eliminated from the study (Table 3). Selected compounds were predicted to be soluble and to possess high gastrointestinal (GI) absorption, which would indicate that they could easily be absorbed through the intestinal tract into the blood stream when orally administered [88] (Table 3). 

In this study, compounds that were predicted to be blood-brain barrier (BBB) permeants were considered for a downstream analysis. Recent studies have shown that most survivors of Ebola infection suffer neurologic complications including seizures, memory loss, headaches, cranial nerve abnormalities, and tremor [89,90]. Ebola has been suggested to cross the brain–blood barrier and may perform a pathogenic role in the onset of encephalitis [91,92]. Ebola has also been reported to exist in some immunologically privileged sites, including the central nervous system (CNS), although the mechanisms through which Ebola affects the CNS remains unclear [90,93]. Potent EBOV drugs with evidence of penetration into the CNS, will therefore be beneficial in the treatment of EVD in the CNS and in other parts of the body. An effective neurologic drug should be able to permeate the blood–brain barrier (BBB) so as to bind to specific receptors and initiate signaling pathways [94]. Therefore, compounds that were not predicted to be able to permeate the BBB were excluded from further analysis. 

Another important parameter considered was the ability of a drug to be eliminated from the central nervous system (CNS) to reduce toxicity in the cells. P-glycoproteins are ATP-dependent efflux transporters extensively distributed and expressed in cells found in the kidney, liver, colon and pancreas [95]. They transport toxins and a wide range of foreign substances including drugs, out of the cell. Consequently, the overexpression of these proteins in diseased cells reduces the chances of successful drug delivery and limits the cellular uptake of drugs from the blood stream into cells [95,96]. As a result, compounds shortlisted in the study were predicted to be P-gp non-substrates (Table 3). 

**Figure 3:**
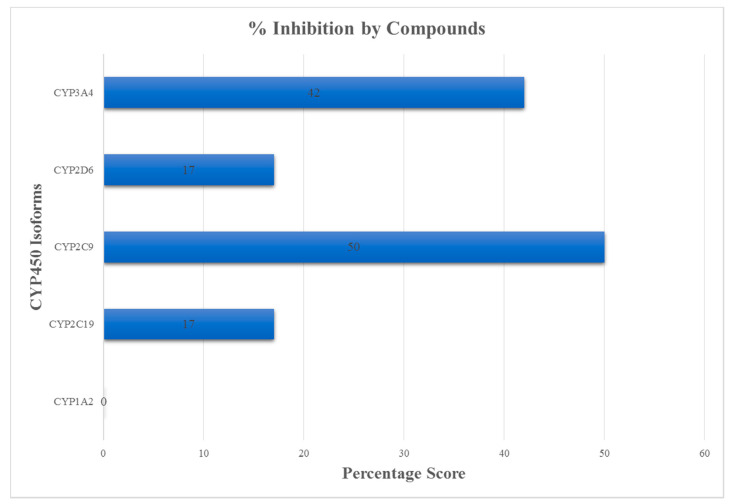
Percentage of inhibition activity by 12 selected compounds against the CYP450 isoforms (namely 1A2, 2C9, 2C19, 2D6 and 3A4). Majority of the compounds inhibited CYP2C9 while CYP1A2 was inhibited by none of the compounds.

Furthermore, the ability of the compounds to inhibit cytochrome (CY) P450 and its essential isoforms, namely 1A2, 2C19, 2C9, 2D6 and 3A4 were assessed. These constitute a superfamily of enzymes that regulate the metabolism and excretion of drugs from the liver [97]. When drugs are co-administered, the inhibition of the activity of these isoforms by one drug may lead to drug interactions and an accumulation of the second drug, resulting in high toxicity levels in targeted cells [80]. Such instances necessitate dosage adjustment or the selection of drugs that do not inhibit the cytochrome P450 system. Approximately 50% and 42% of the compounds were predicted to be inhibitors of 2C9 or 3A4, respectively (Figure 3). Interestingly, none of these compounds were predicted to inhibit 1A2, with only 17% acting as inhibitors of both 2C19 and 2D6. In comparison, the known inhibitors were predicted to be inhibitors of more than half the number of the CYP450 enzymes (Supplementary Table S1). Therefore, compounds that were non-substrate to at least 3 of the cytochrome isoforms (1A2, 2C19, 2C9, 2D6 and 3A4) were shortlisted.

Eleven of the compounds were predicted to be non-mutagenic while ten were neither tumorigenic nor reproductive, and nine compounds were shown to be irritants (Table 4). Amodiaquine was predicted to be highly mutagenic, irritant and to produce a reproductive effect, while Chloroquine was also highly mutagenic and irritant. Gossypetin was also predicted to be highly mutagenic, while EGCG and taxifolin were predicted to be non-toxic. Overall, the majority of the potential lead compounds showed a lower possibility of toxicity as compared to amodiaquine and chloroquine. Early toxicity profiling during in silico studies allows for the prioritization of compounds with desirable properties and a low toxicity risk [98]. Twelve compounds were selected after the ADMET screening for further analysis.

### 3.4. Molecular Interactions of Protein-Ligand Complexes 

**Figure 4:**
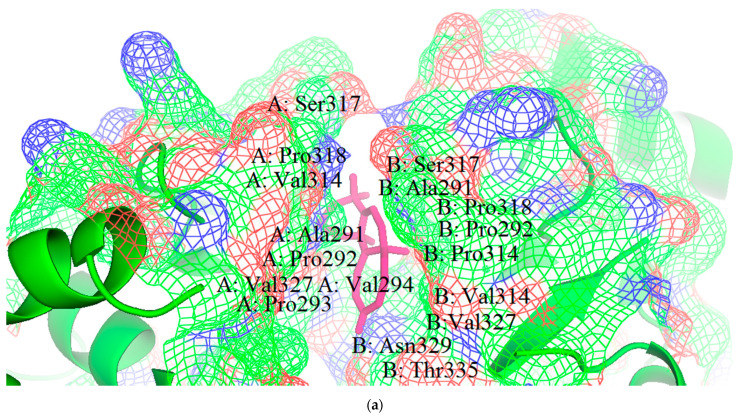

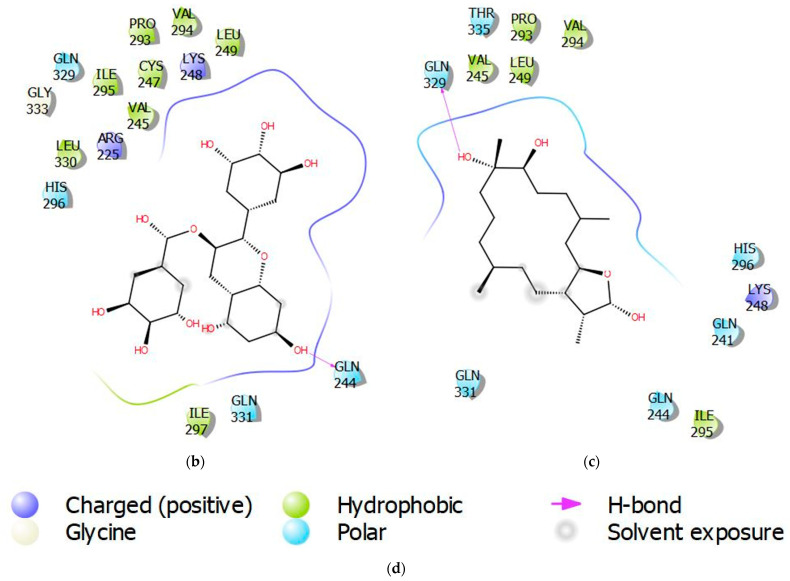
(**a**) A cartoon diagram of EBOV VP35 in complex with ZINC000095486250 depicting the mesh representation of the binding pocket, (**b**) 2D representation of EGCG showing molecular interaction with VP35. The hydrogen bond formed with Gln244 is colored purple, (**c**) 2D representation of NANPDB86 showing molecular interaction with VP35. The hydrogen bond formed with Gln329 is colored purple and (**d**) the legend for the protein-ligand interaction profiles.

Hydrogen and hydrophobic interactions were formed between the ligands and the amino acid residues, within the active sites of EBOV VP35 (Figure 4 and Supplementary Figure S2). The amino acids involved in the interactions and the bond lengths were also determined (Table 2). A previous study virtually screened compounds obtained via pharmacophore modelling against VP35 [99], in which the top 7 compounds were observed to interact with residues Gln241, Lys248, Ile295, Ile303, Pro304 and Phe328 [99]. NANPDB86 formed one hydrogen bond with Gln329 (bond length 2.0 Å) and hydrophobic interactions with Val245, Leu249, Pro293, Val294 and Ile295 (Figure 4). NANPDB95 also formed hydrophobic interactions with Pro316, Ala291, Pro292, Leu249, Pro293, Val294, Val327, Ile286, Ala290, Pro315, Pro318, and Val314 (Supplementary Figure S2). Similarly, NANPDB142 formed hydrophobic interactions with Pro318, Ala291, Pro315, Pro316, Ala290, Val294, Val327, Val314, and Leu249 (Supplementary Figure S2). NANPDB205 interacted with Leu249, Pro293, Val245, and Ile295 via hydrophobic bonds (Supplementary Figure S2). NANPDB397 formed hydrophobic interactions with Pro318, Val314, Ala291, Pro292, Pro293, Val327, and Val294 (Supplementary Figure S2). NANPDB2412 formed hydrophobic bonds with Pro318, Pro316, Ala290, Pro315, Ala291, Val314, Pro292, Val294, Pro293, and Val327 (Supplementary Figure S2). Additionally, NANPDB2476 formed hydrophobic bonds with Pro316, Ala291, Pro315, Pro318, Pro292, Val314, Val327 and Val294 (Supplementary Figure S2). NANPDB3355 formed hydrophobic bonds with Pro316, Ala290, Ala291, Pro292, Val314, Pro318, Val294 and Val327 (Supplementary Figure S2). NANPDB4048 also interacted with Pro318, Ala291, Val314, Pro292, Pro293, Leu249, Val294 and Val327 through hydrophobic interactions (Supplementary Figure S2). ZINC000014612849 formed hydrophobic interactions with Val314, Pro292, Ala291, Pro318, Pro315, Val327 and Val294 (Supplementary Figure S2). ZINC000033831303 formed hydrophobic interactions with Pro293, Leu249, Ile295, Val245 and Val294 (Supplementary Figure S2). ZINC000095486250 interacted with Ala291, Pro318, Pro292, Val314, Pro293, Val327 and Val294 via hydrophobic interactions (Supplementary Figure S2). Interestingly, the 12 hits interacted with amino acid residues Val245, Leu249, Pro293, Val294, Ile295, Pro316, Ala291, Gln329, Pro292, Leu249, Val327, Ile286, Ala290, Pro315, Pro318 and Val314, which were in pocket 1 of EBOV VP35 (Table 1). Similarly, amodiaquine, chloroquine, EGCG, gossypetin and taxifolin formed intermolecular bonds with the active site residues Gln244, Val245, Leu249, Ala290, Ala291, Pro292, Pro293, Val294, Ile295, Ile297, Val314, Pro315, Pro318, Val327 and Leu330, which are present in pocket 1 (Table 1) except Cys247. Amodiaquine has previously been shown in an in silico study to interact with residues Ile295, Lys248 and Gln244 which favour amodiaquine binding to the VP35 [27]. Similarly, tetrahydrocurcumin, curcumin and demethoxycurcumin were reported to interact with Gln244, Leu249, Pro293, Val294, Pro316, Val327 and Gln329 [100], found in pocket 1 of EBOV VP35 (Table 1). Molludistin was shown to interact with Gln329 and Leu330 while Xanthomicrol interacts with Gln244 and Val294 [81]. These residues interacting with the known inhibitors also formed intermolecular bonds with the 12 hits. However, from the results, Val245, Leu249, Ala290, Ala291, Pro292, Val294, Ile295, Val314, Pro315, Pro318 and Val 327 interacted with all ligands that bind in pocket 1, and could therefore be investigated as potential critical residues essential for inhibition [78,80].

### 3.5. Biological Activity Predictions for Ligands

The biological activities of the selected molecules were predicted using Prediction of Activity Spectra for Substances (PASS), which is built using a naïve Bayesian classifier. The training dataset comprises of data from several sources such as chemical databases, publications and patents resulting in over 26,000 biological compound-including leads, drug-like compounds, toxic substances and FDA-approved drugs [101]. PASS uses the 2D structural formula of the compound as the input data to predict the biological activity of the compound using position-specific descriptors at an average accuracy of 95% [102]. The ability of a potential drug lead to inhibit the synthesis of EBOV RNA and the proteins essential for viral replication is imperative in the identification of therapeutic agents for the treatment of EVD [103,104]. The compounds in this study were predicted to be inhibitors of DNA polymerase I, synthesis EBOV proteins, RNA and transcription factors with the exception of NANPDB397 and ZINC000014612849 (Table 5). 

Additionally, NANPDB4048 was predicted to be a membrane permeability inhibitor with a Pa of 0.753 and Pi of 0.020, and therefore warrants pharmacological investigation to evaluate its potential to prevent the invasion of EBOV into host cells [105,106]. NANPDB4048 demonstrated the highest Pa value of 0.753 as a membrane permeability inhibitor while NANPDB142 recorded the lowest Pi value of 0.003 with a Pa of 0.625 as a DNA polymerase I inhibitor (Table 5). Furthermore, the compounds selected in this study were compounds that possessed probable activity (Pa) values greater than their probable inactivity (Pi) [107], reinforcing the need for the in vitro testing of their anti-EBOV activity [108]. Additionally, the predicted anti-EBOV inhibition efficiency values (IC_50_), using a random forest-based classifier [60] for NANPDB2412, NANPDB2476, NANPDB4048 and ZINC000095486250 were obtained as 11.48, 8.83, 3.35 and 11.99 μM, respectively.

### 3.6. Assessment of Quality of Ligands

The quality of the ligands was evaluated using metrics such as the ligand efficiency (LE), fit quality (FQ), inhibitory constant (Ki), LE-dependent lipophilicity (LELP) and LE_scale (Table 6). Ligand efficiency metrics have long been used as a criteria to identify plausible compounds during hit-to-lead optimization [63,109]. These indices have been widely applied in many studies to distinguish between promising and non-promising compounds [110]. The average LE value for lead-like compounds should be at least 0.30 kcal/mol/HA [111]. The LE values obtained ranged from 0.32 to 0.42 (Table 6), which were above the values of other compounds with similar number of heavy atoms [86] and also the proposed LE value. 

The other useful parameters for evaluating the physicochemical properties of compounds were the LE-dependent lipophilicity (LELP) and LE_scale metrics. An increase in the lipophilicity of a compound is likely to result in multiple targets binding, which is undesirable [112,113]. A study has demonstrated that increasing the molecular weight (MW) generally leads to the deterioration of the ADMET parameters [56]. Given that LE is size dependent, a scaling function known as LE_scale is introduced to address the limitations of LE [114]. The LE_scale and LELP values ranged from 0.39 to 0.40 and 4.36 to 10.11, respectively (Table 6). The suggested values of LELP range from −10 to 10, and compounds that follow the RO5 possess LELP values less than 16.5; therefore, the drug leads fell within an acceptable range [115]. 

An FQ index is also applied to normalize LE across a wide range of molecular sizes and allows for a size-independent comparison of compounds [116]. An FQ value is a plausible measure of efficiency in the lead optimization process [117]. An FQ of close to 1 indicates the optimal binding of a compound [108]. The twelve compounds had FQ scores close to 1, with the highest value of 0.89 and lowest value of 0.82 suggesting optimal ligand–protein binding. Similar FQ values were reported in a recent study, with the lowest recorded value of 0.8 and highest recorded value of 0.9 [86]. Lastly, the potency of a drug is dependent on its inhibitory constant (Ki), whereby the lower the Ki, the more likely the drug is to inhibit the target protein [62]. In this study, the calculated Ki values ranged from 0.97 μM to 1.37 μM (Table 6), which shows a good inhibitory potential of the molecules. Additionally, the Ki values of the ligands were close to those reported in previous studies [86,114]

### 3.7. Molecular Dynamics Simulation of VP35-Ligand Complexes

**Figure 5:**
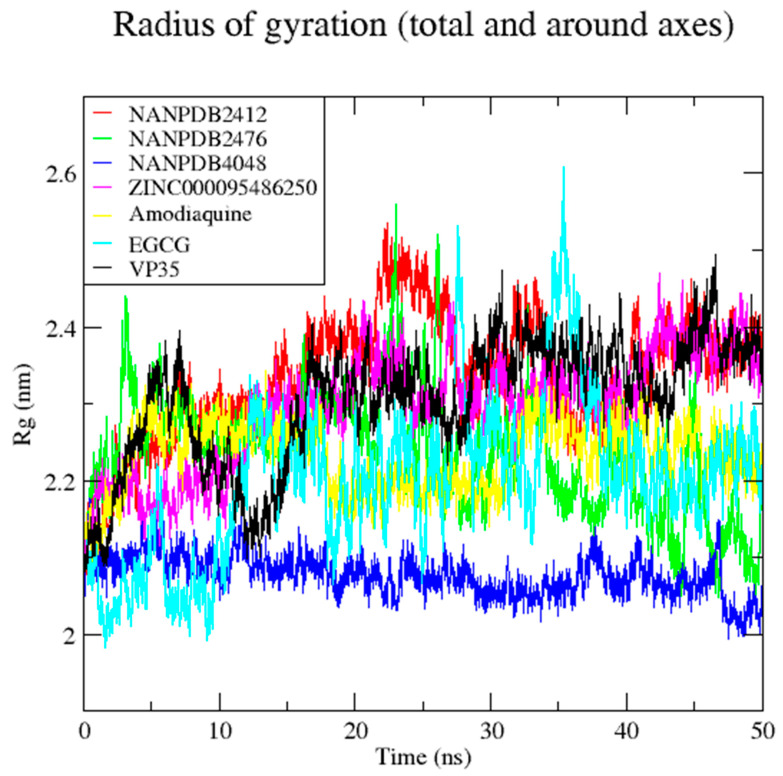
The time evolution of radius of gyration (Rg) for the EBOV VP35-complexes: Amodiaquine, EGCG, NANPDB4048, NANPDB2412, NANPDB2476, ZINC000095486250 and VP35 are indicated in yellow, cyan, blue, red, green, magenta and black, accordingly.

Complexes of four compounds and of two known inhibitors of EBOV VP35 protein were subjected to MD simulations over a 50 ns period to understand their interaction pattern and dynamic behavior [65]. The radius of gyration (Rg) of the protein structure determines its compactness and, therefore, a protein that is stably folded is likely to remain steady over a period of time [118]. The Rg values obtained from the MD analysis revealed that the VP35-NANPDB4048 complex was very stable and compact over the period of 50 ns with an average Rg of 2.08 nm (Figure 5). The VP35-NANPDB2412 complex showed a gradual increase in Rg from 0 to about 25 ns, with peaks recorded at around 25 ns which remained steady throughout the 50 ns, reaching an average Rg value of 2.31 nm. The VP35-NANPDB2476 complex experienced a small fluctuation between 0 and 35 ns, with an average Rg of 2.31 nm, and gradually fell from a value of 2.23 to 2.08 nm. The VP35-ZINC000095486250 complex increased in Rg, from 0 to around 20 ns and maintained a steady stability to 50 ns with an average 2.28 nm. The VP35-Amodiaquine complex experienced few fluctuations, with an average Rg of 2.21 nm, and was stable from 16 to around 32 ns. Lastly, the VP35-EGCG complex experienced little fluctuations with peaks recorded at around 26 and 36 ns, and the average Rg observed was 2.25 nm. Any discrepancies that exists for the stability of the complexes may be attributed to the sensitivity of the complex to thermodynamic properties such as high temperatures and pressures, causing the protein to unfold [119]. When the radius of gyration is higher, it affects the compactness of the protein–ligand complex, resulting in weak interactions between the protein and the ligand [120].

**Figure 6:**
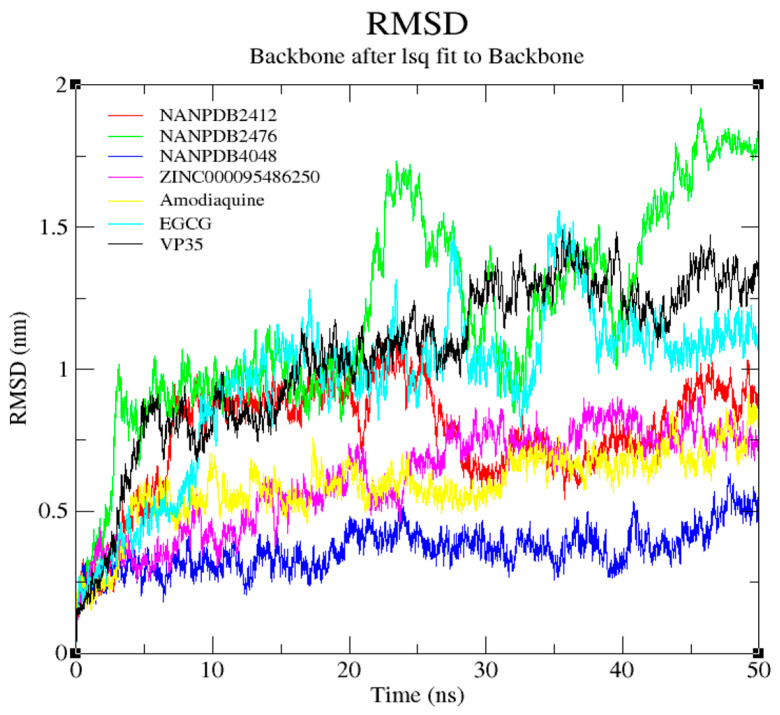
Backbone RMSD of the EBOV VP35-complexes: Amodiaquine, EGCG, NANPDB4048, NANPDB2412, NANPDB2476, ZINC000095486250 and VP35 are indicated in yellow, cyan, blue, red, green, magenta and black, accordingly.

Additionally, the stability of the docked complexes was analyzed using the RMSD generated from the MD simulations. The backbone of the VP35-NANPDB4048 complex was more stable than the rest of the complexes (Figure 6), with a small deviation as it increased from 0.05 nm to 0.65 nm over 50 ns with an RMSD value of around 0.45 nm. The RMSD of VP35-NANPDB2412 complex gradually rose from 0 to around 0.95 nm for a period of 8 ns from the onset and remained steady until approximately 20 ns, thereafter it experienced some fluctuations in stability over the rest of the period with a deviation of about 0.8 nm. The EBOV VP35-NANPDB2476 complex experienced a sharp rise in RMSD from 0 to around 5 ns and remained steady for over 15 ns. It displayed a significant increase in RMSD during the remaining 30 ns, with a deviation of approximately 1.3 nm. The backbone of the VP35-ZINC000095486250 complex was relatively stable with little deviations. Its RMSD steadily increased with a deviation of 0.7 nm from 0.1 to 0.85 nm for around 40 ns and declined gradually until the end of the 50 ns period. Initially, the VP35-Amodiaquine complex had a steep slope from about 0.5 to 0.58 nm at around 5 ns after which it decreased gradually until the end of the 50 ns period, with a deviation of approximately 0.69 nm. The VP35-EGCG complex experienced unstable RMSD values with peaks recorded at around 1.62 nm and 1.73 nm around 27 ns and 36 ns, respectively showing a deviation of about 1.35 nm. Overall, VP35-NANPDB4048 and VP35-ZINC000095486250 complexes showed more stability than the known inhibitors in this study. This implies that the deviations of both complexes were relatively low, demonstrating their stability [121]. Moreover, since all the complexes depicted an average deviation below the similarity threshold of 2 Å, it is not likely that any significant conformational changes in the structure of the ligands could have occurred, although the structural integrity of the protein may have been affected [108,122].

### 3.8. MM-PBSA Computations on Potential Lead Compounds

The MM-PBSA method was used to determine the binding free energies of the docked ligands. The selected compounds possessed low binding free energies (Table 7). Similarly, the standard compounds, amodiaquine and EGCG all had low binding energies of −92.4 kJ/ mol and −44.564 kJ/ mol, respectively (Table 7) signifying very strong binding to the VP35 protein. The amodiaquine inhibited EBOV replication, without significant toxicity, with an IC_50_ of 1.45 µM [123]. Among all the compounds, ZINC000095486250 possessed the minimum binding free energy of −94.213 kJ/mol. The total binding energies were highly negative due to the contributions from van der Waals energy, SASA energy, electrostatic energy and polar solvation energy [124] (Table 7). However, the van der Waals energy contributed significantly to the low total binding energy. Although, the binding energy was considerably reduced by the SASA and electrostatic interactions, it was regulated by a rather stronger unfavorable polar solvation energy [125] (Table 7). The SASA energy term estimates the interactions between the complexes and solvents. The EBOV VP35-ligand complexes possessed SASA energy values ranging from −10.531 to −18.495 kJ/mol (Table 7). EBOV VP35-Amodiaquine possessed the lowest SASA energy, at −18.495 kJ/mol while EBOV VP35-NANPDB2476 demonstrated the highest SASA energy of −10.531 kJ/mol. All the EBOV VP35-ligand complexes demonstrated very low van der Waals interactions ranging from −72.353 kJ/mol to −150.934 kJ/mol (Table 7). Interestingly, EBOV VP35-Amodiaquine demonstrated the lowest van der Waals energy, of −150.934 kJ/mol, while EBOV VP35-NANPDB2476 showed the highest van der Waals energy of −72.353 kJ/mol (Table 7).

**Figure 7:**
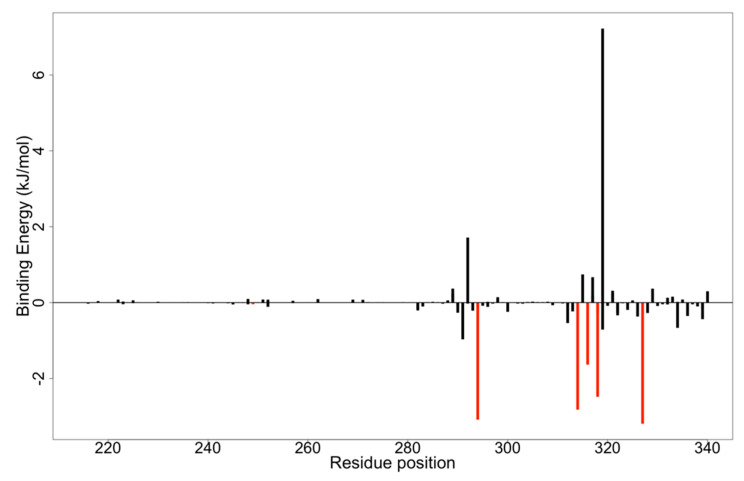
A plot of the binding free energy contribution per residue of EBOV VP35-NANPDB2476 complex derived from molecular mechanics Poisson-Boltzmann surface area (MM-PBSA) analysis. The critical residues contributing to the binding energies are shown in red.

The molecular docking simulations showed ZINC000095486250 had a binding affinity of −8.1 kcal/mol (−33.8904 kJ/mol), which is the same as that of EGCG. However, EGCG had a binding free energy of −44.564 kJ/mol. Hence, the significance of MM-PBSA calculations, used to reinforce the scoring functions of the virtual screening, becomes evident [126]. The MM-PBSA analysis suggested that residues involved in hydrophobic interactions are, most importantly, involved in protein-ligand binding. The residues with energy values less than −5 kJ/ mol or greater than 5 kJ/mol are more likely to contribute to the overall binding free energies associated with the protein-ligand interactions (Figure 7 and Supplementary Figure S3) [127]. Residues which contribute high positive energies are unfavourable for ligand binding, whereas residues that contribute highly negative energies are favourable for ligand binding [128]. The per-residue decomposition results suggested that residues located in pocket 1 including Leu249, Val294, Val314, Pro316, Pro318 and Val327 (Table 1) could play critical roles in the binding mechanisms (Supplementary Figure S3). Thus, it is strongly recommended that in the design and development of novel EBOV VP35 inhibitors, these critical residues should be accorded adequate consideration. Furthermore, any undesirable residues could be ignored [127] in favor of the critical residues.

### 3.9. Structural Similarity Search of Hits

The four compounds that were identified as hits were subjected to structural similarity searches via DrugBank (Table 8). Peridinin was shown to be structurally similar to NANPDB2476, with a similarity score of 0.717. Interestingly, peridinin has been reported to strongly exhibit anti-dengue virus activity for all the serotypes of dengue virus [129]. Peridinin was found to have IC_50_ values of 7.62, 4.50, 5.84 and 6.51 μg/mL against DENV-1, DENV-2, DENV-3 and DENV-4, respectively [129]. Peridinin, extracted from *Isis hippuris* has also been reported to inhibit Human T-cell leukemia virus type 1 (HTLV-1)-infected T-cell lines [130].

NANPDB2412 was found to be structurally similar to carbenoxolone with a similarity score of 0.842. Carbenoxolone has been shown to possess broad-spectrum virucidal activity against various viruses including the Dengue, herpes and vaccinia viruses [131,132,133]. Carbenoxolone was reported to reduce DENV-2 mRNA expression by 5- and 10-fold at concentrations of 50 and 100 µM, respectively [131]. 

**Table 8:** List of two-dimensional structures of potential lead compounds obtained using Marvin Sketch (ChemAxon Ltd., MarvinSketch version 17.28.00, Budapest, Hungary) [134].

Compound ID	IUPAC Names	Two-Dimensional Structure
NANPDB2412	(1R,2R,5S,7S,8S,13R,14R,17R)-2,7,14-trimethyl-16-oxapentacyclo[9.7.0.0^2^,^8^.0^5^,^7^.0^13^,^17^]octadeca-3,10-diene-12,15-dione	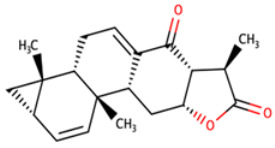
NANPDB2476	(1S,3R,10S,11R,14R,16R)-5,11,14-trimethyl-2,7-dioxapentacyclo[8.8.0.0¹,³.0⁴,⁸.0^11^,^16^]octadeca-4,8-dien-6-one	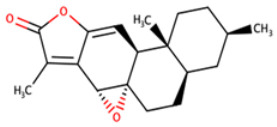
NANPDB4048	(1Z,2S,3aR,3bS,9aR,9bS,11aS)-1-ethylidene-2-hydroxy-9a,11a-dimethyl-1H,2H,3H,3aH,3bH,4H,5H,7H,8H,9H,9aH,9bH,11aH-cyclopenta[a]phenanthren-7-one	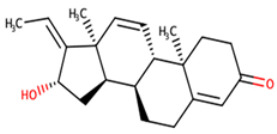
ZINC000095486250	(6aR,12aS)-6a,9,9,12a-tetramethyl-3H,4H,5H,6aH,7H,9H,10H,11H,12H,12aH-naphtho[2,1-b]oxocin-3-one	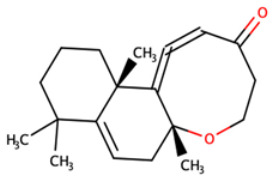

## 4. Implications and Future Prospects

Natural products have been found to act effectively against a wide range of diseases; however, they are underutilized in the search for anti-EBOV therapeutic agents. Thus, this study complements recent efforts in identifying novel EBOV inhibitors [99,108,135,136,137,138]. Such previous studies have led to the discovery of three Indonesian natural compounds which have been found to be active against EBOV VP335, namely myricetin 3-robinobioside, theasaponin and kaempferol 3-(6G-malonylneohesperidoside) [138]. Phytochemicals belonging to the genus *Ocinum,* were also screened against EBOV VP35 using an in silico approach. Isovitexin, cosmosiin and molludistin were suggested to be the best compounds among the phytochemicals, possessing desirable properties against EBOV VP35 [81]. The drug-like compounds identified for use against EBOV VP35 were formulated using thorough computational approaches and could be used as model structures for further optimization and synthesis in experimental characterization.

## 5. Conclusions

This study effectively applied in silico drug discovery techniques to identify potential anti-EBOV molecules from the African flora. These compounds include NANPDB2412, NANPDB2476, NANPDB4048 and ZINC000095486250. They were physicochemically screened and determined to be drug-like with a low predicted toxicity risk. They were predicted to display antiviral biological activity, and to possess reasonably good inhibition efficiency and Ki values. The study predicted novel binding mechanisms, including critical residues essential for biomolecular interactions. A structural similarity search also revealed that NANPDB2476 and NANPDB2412 are structurally similar to peridinin and carbenoxolone with similarity scores of 0.717 and 0.842, respectively. Peridinin and carbenoxolone have previously been shown to possess virucidal activity, which reinforces the necessity of an antiviral potential for the compounds. These drug-like compounds are promising potential leads, which warrant further in vitro experimentation to corroborate their predicted antiviral bioactivity. Fragments of the identified molecules could be optimized as novel inhibitors to aid the synthesis of anti-EBOV agents.

## Figures and Tables

**Table 1:** Major binding sites of EBOV VP35 predicted using CASTp and the amino acid residues within each binding pocket. SA: Solvent accessible.

Binding Sites	Chain	Amino Acid Residues	Surface Area (SA)/Å^2^	Volume/Å^3^
Pocket 1	A	Val245, Lys248, Leu249, Asp252, Ser253, Ile286, Phe287, Gln288, Asp289, Ala290, Ala291, Pro292, Pro293, Val294, Ile295, His296, Ile297, Arg298, Val314, Pro315, Pro316, Ser317, Pro318, Lys319, Val327, Gln329, Leu330, Gln331, Gly333, Thr335.	1155.05	1078.689
	B	Gln241, Gln244, Val245, Lys248, Leu249, Asp252, Ser253, Ile286, Gln288, Asp289, Ala290, Ala291, Pro292, Pro293, Val294, Ile295, His296, Val314, Pro315, Pro316, Ser317, Pro318, Lys319, Val327, Gln329, Leu330, Gln331, Gly333, Lys334, Thr335.		
Pocket 2	B	Asp218, Ile219, Asn254, Leu256, Asp257	48.092	35.5916
Pocket 3	A	Asp218, Ile219, Asn254, Leu256, Asp257	52.040	34.782

**Table 2:** The binding energies of selected compounds and known inhibitors as well as their intermolecular bonds with EBOV VP35.

Compound ID	Binding Energy (kcal/mol)	Number of Hydrogen Bonds	Hydrogen Bond Residues	Hydrogen Bond Length (Å)	Hydrophobic Contacts
NANPDB86	−8.5	1	Gln329	2.0	Val245, Leu249, Pro293, Val294, Ile295
NANPDB95	−8.1	0	-	-	Pro316, Ala291, Pro292, Leu249, Pro293, Val294, Val327, Ile286, Ala290, Pro315, Pro318, Val314
NANPDB142	−8.0	0	-	-	Pro318, Ala291, Pro315, Pro316, Ala290, Val294, Val327, Val314, Leu249
NANPDB205	−8.3	0	-	-	Leu249, Pro293, Val245, Ile295
NANPDB397	−8.1	0	-	-	Pro318, Val314, Ala291, Pro292, Pro293, Val327, Val294
NANPDB2412	−8.2	0	-	-	Pro318, Pro316, Ala290, Pro315, Ala291, Val314, Pro292, Val294, Pro293, Val327
NANPDB2476	−8.0	0	-	-	Pro316, Ala291, Pro315, Pro318, Pro292, Val314, Val327, Val294
NANPDB3355	−8.2	0	-	-	Pro316, Ala290, Ala291, Pro292, Val314, Pro318, Val294, Val327
NANPDB4048	−8.2	0	-	-	Pro318, Ala291, Val314, Pro292, Pro293, Leu249, Val294, Val327
ZINC000014612849	−8.1	0	-	-	Val314, Pro292, Ala291, Pro318, Pro315, Val327, Val294
ZINC000033831303	−8.0	0	-	-	Pro293, Leu249, Ile295, Val245, Val294
ZINC000095486250	−8.1	0	-	-	Ala291, Pro318, Pro292, Val314, Pro293, Val327, Val294
Amodiaquine	−7.0	0	-	-	Ala291, Pro318, Ala291, Pro315, Val327, Val294, Pro292, Val314
Chloroquine	−5.9	0	-	-	Pro318, Val314, Val327, Pro292, Ala291, Val294, Pro293
EGCG	−8.1	1	Gln244	2.01	Val294, Pro293, Leu249, Val245, Cys247, Ile297, Leu330
Gossypetin	−7.5	1	Leu330	1.97	Ile295, Val294, Pro293, Leu249, Val245
Taxifolin	−7.4	0	-	-	Val314, Ala290, Ala291, Pro318, Val294, Val327, Pro292, Leu249

**Table 3:** Pharmacokinetic profile of the top 12 compounds and 5 known EBOV VP35 inhibitors comprising of Estimated Solubility (ESOL). Blood Brain Barrier (BBB), Gastrointestinal (GI) and P-glycoprotein (Pgp).

Compound ID	Estimated Solubility Log S	Estimated Solubility Class	GI Absorption	BBB Permeant	P-glycoprotein Substrate
NANPDB86	−3.79	Soluble	High	Yes	No
NANPDB95	−3.57	Soluble	High	Yes	No
NANPDB142	−3.77	Soluble	High	Yes	No
NANPDB205	−2.61	Soluble	High	Yes	No
NANPDB397	−3.09	Soluble	High	Yes	No
NANPDB2412	−3.99	Soluble	High	Yes	No
NANPDB2476	−3.89	Soluble	High	Yes	No
NANPDB3355	−3.25	Soluble	High	Yes	No
NANPDB4048	−3.73	Soluble	High	Yes	No
ZINC000014612849	−3.00	Soluble	High	Yes	No
ZINC000033831303	−3.89	Soluble	High	Yes	No
ZINC000095486250	−3.41	Soluble	High	Yes	No
Amodiaquine	−5.9	Moderately soluble	High	Yes	No
Chloroquine	−4.55	Moderately soluble	High	Yes	No
EGCG	−3.56	Soluble	Low	No	No
Gossypetin	−3.40	Soluble	Low	No	No
Taxifolin	−2.66	Soluble	High	No	No

**Table 4:** Toxicity profiles of selected compounds and known inhibitors. The profiles consist of mutagenicity, tumorigenicity, reproductive effect and irritancy.

Compound ID	Mutagenic	Tumorigenic	Reproductive Effect	Irritant
NANPDB86	None	None	None	None
NANPDB95	None	None	None	None
NANPDB142	None	None	None	None
NANPDB205	None	None	High	None
NANPDB397	None	None	None	None
NANPDB2412	None	None	None	None
NANPDB2476	None	None	None	High
NANPDB3355	None	High	None	High
NANPDB4048	None	None	High	None
ZINC000014612849	Low	None	None	None
ZINC000033831303	High	High	None	High
ZINC000095486250	None	None	None	None
Amodiaquine	High	None	High	High
Chloroquine	High	None	None	High
EGCG	None	None	None	None
Gossypetin	High	None	None	None
Taxifolin	None	None	None	None

**Table 5:** The anti-viral activity prediction of the selected compounds.

Compound ID	Biological Activity	Pa	Pi
NANPDB86	Rhinovirus	0.444	0.052
	Herpes	0.334	0.069
	Protein synthesis inhibitor	0.467	0.008
	Transcription factor inhibitor	0.39	0.026
	RNA synthesis inhibitor	0.287	0..63
NANPDB95	Herpes	0.394	0.038
	Picornavirus	0.337	0.173
	Transcription factor inhibitor	0.557	0.008
	Protein synthesis inhibitor	0.493	0.007
	RNA synthesis inhibitor	0.331	0.038
NANPDB142	Rhinovirus	0.413	0.078
	Herpes	0.332	0.071
	Picornavirus	0.352	0.156
	DNA polymerase 1 inhibitor	0.625	0.003
	RNA synthesis inhibitor	0.285	0.065
NANPDB205	Adenovirus	0.222	0.176
	Protein synthesis inhibitor	0.238	0.041
	RNA synthesis inhibitor	0.251	0.100
	DNA synthesis inhibitor	0.207	0.141
NANPDB397	-	-	-
NANPDB2412	Herpes	0.410	0.031
	Rhinovirus	0.345	0.167
	Transcription factor inhibitor	0.283	0.013
	DNA synthesis inhibitor	0.232	0.101
	RNA synthesis inhibitor	0.231	0.125
NANPDB2476	Influenza	0.476	0.027
	Rhinovirus	0.381	0.114
	Protein synthesis inhibitor	0.376	0.019
	RNA synthesis inhibitor	0.277	0.072
NANPDB3355	Rhinovirus	0.552	0.012
	Protein synthesis inhibitor	0.353	0.022
	Transcription factor inhibitor	0.240	0.093
	RNA synthesis inhibitor	0.241	0.111
NANPDB4048	Influenza	0.621	0.011
	Rhinovirus	0.362	0.140
	Membrane permeability inhibitor	0.753	0.020
	RNA synthesis inhibitor	0.484	0.009
ZINC000014612849	-	-	-
ZINC000033831303	RNA synthesis inhibitor	0.281	0.069
ZINC000095486250	Influenza	0.399	0.047
	Herpes	0.273	0.111
	RNA synthesis inhibitor	0.298	0.056
	DNA polymerase I inhibitor	0.275	0.098
Amodiaquine	-	-	-
Chloroquine	-	-	-
EGCG	Influenza	0.771	0.003
	Rhinovirus	0.514	0.020
	Herpes	0.480	0.012
	HIV	0.300	0.008
	Hepatitis B	0.316	0.029
	Transcription factor inhibitor	0.404	0.007
	RNA synthesis inhibitor	0.318	0.044
	DNA polymerase I inhibitor	0.294	0.070
Gossypetin	Hepatitis B	0.498	0.005
	Influenza	0.415	0.042
	Membrane permeability inhibitor	0.953	0.002
	RNA synthesis inhibitor	0.358	0.029
	DNA polymerase I inhibitor	0.331	0.040
Taxifolin	Influenza	0.620	0.011
	Herpes	0.492	0.010
	Rhinovirus	0.503	0.023
	Hepatitis B	0.399	0.015
	Membrane permeability inhibitor	0.850	0.005
	Transcription factor inhibitor	0.413	0.022
	DNA polymerase I inhibitor	0.329	0.041
	RNA synthesis inhibitor	0.394	0.021

**Table 6:** The Ligand metrics used to evaluate the quality of the selected compounds, namely ligand efficiency (LE), fit quality (FQ), LE_scale, LE-dependent lipophilicity (LELP) and inhibitory constant (Ki).

Compound ID	Number of Heavy Atoms	Log P	Ki	LE	LE_Scale	FQ	LELP
NANPDB86	24	2.79	5.87 × 10^−7^	0.354	0.404	0.876	7.88
NANPDB95	24	2.94	1.15 × 10^−6^	0.338	0.404	0.837	8.70
NANPDB95	24	2.94	1.15 × 10^−6^	0.338	0.404	0.837	8.70
NANPDB142	25	2.93	1.37 × 10^−6^	0.320	0.391	0.818	9.16
NANPDB205	20	1.81	8.23 × 10^−7^	0.415	0.467	0.889	4.36
NANPDB397	24	2.66	1.15 × 10^−6^	0.338	0.404	0.837	7.87
NANPDB2412	23	3.25	9.74 × 10^−7^	0.357	0.418	0.854	9.10
NANPDB2476	22	3.55	1.37 × 10^−6^	0.364	0.433	0.841	9.75
NANPDB3355	24	2.43	9.74 × 10^−7^	0.342	0.404	0.847	7.11
NANPDB4048	23	3.61	9.74 × 10^−7^	0.357	0.418	0.854	10.11
ZINC000014612849	25	2.22	1.15 × 10^−6^	0.324	0.391	0.829	6.85
ZINC000033831303	23	3.37	1.37 × 10^−6^	0.348	0.418	0.833	9.68
ZINC000095486250	21	3.68	1.15 × 10^−6^	0.386	0.449	0.860	9.53

**Table 7:** The free energy terms for the binding of compounds to the EBOV VP35 protein. The energy values are presented as average ± standard deviations in kJ/mol. The binding affinity scores from the docking studies are represented as “kcal/mol (kJ/mol)”, where the calculated binding affinity in kJ/mol are presented in brackets.

Compound ID	Binding Affinity from Docking [kcal/mol (kJ/mol)]	van der Waal Energy (kJ/mol)	Electrostatic Energy (kJ/mol)	Polar Solvation Energy (kJ/mol)	SASA Energy (kJ/mol)	Binding Energy (kJ/mol)
NANPDB2412	−8.2 (−34.3088)	−112.794 ± 31.343	−4.338 ± 7.888	63.305 ± 25.933	−13.955 ± 3.243	−67.782 ± 17.041
NANPDB2476	−8.0 (−33.472)	−72.353 ± 15.702	−8.393 ± 9.299	46.887 ± 21.330	−10.531 ± 2.288	−44.390 ± 19.503
NANPDB4048	−8.2 (−34.3088)	−122.063 ± 24.789	−3.170 ± 8.186	68.675 ± 23.656	−15.854 ± 2.967	−72.413 ± 15.915
ZINC000095486250	−8.1 (−33.8904)	−133.848 ± 15.162	−6.489 ± 7.863	62.413 ± 10.653	−16.289 ± 1.014	−94.213 ± 12.755
Amodiaquine	−7.0 (−29.288)	−150.934 ± 19.558	−6.282 ± 8.679	83.311 ± 13.703	−18.495 ± 1.647	−92.400 ± 15.855
EGCG	−8.1 (−33.8904)	−110.393 ± 27.459	−46.227 ± 20.847	126.216 ± 35.236	−14.160 ± 3.019	−44.564 ± 23.104

## Data Availability

Not applicable.

## Supplementary Materials

The following are available online at https://www.mdpi.com/article/10.3390/biomedicines9121796/s1, Figure S1: Receiver Operator Characteristic (ROC) curve generated after screening active molecules and their decoys against EBOV VP35 protein. The AUC value for the curve was 0.72, indicating the validity of the classification, Figure S2: 2D diagrams of VP35-ligand complexes showing the hydrogen and hydrophobic interactions with the amino acid residues involved. The hydrogen bond with Gln329 is colored purple. The 2D protein-ligand interaction profile of EBOV VP35 in complex with (A) NANPDB86, (B) NANPDB95, (C) NANPDB142, (D) NANPDB205, (E) NANPDB397, (F) NANPDB2412, (G) NANPDB2476, (H) NANPDB3355, (I) NANPDB4048, (J) ZINC000014612849, (K) ZINC000033831303, (L) ZINC000095486250, (M) Amodiaquine, (N) Chloroquine, (O) EGCG, (P) Gossypetin, and (Q) Taxifolin, Figure S3: Molecular mechanics Poisson-Boltzmann surface area (MM-PBSA) plot of binding free energy contribution per residue of the VP35-ligand complexes: (A) NANPDB2412, (B) NANPDB2476, (C) NANPDB4048, (D) ZINC000095486250, (E) Amodiaquine, and (F) EGCG Fluctuations by predicted critical residues are shown in red., Table S1: ADME prediction results of 12 compounds and 5 known inhibitors for Cytochrome P450 (CYP) inhibition.
