# Supplementary material for: Computational Study on Potential Novel Anti-Ebola Virus Protein VP35 Natural Compounds
Source: Biomedicines. 2021 Nov 30;9(12):1796. doi: 10.3390/biomedicines9121796 (PMC8698941; doi:10.3390/biomedicines9121796)
Supplement: Supplementary file 1 [file biomedicines-09-01796-s001.zip › biomedicines-1417001-supplementary.pdf]

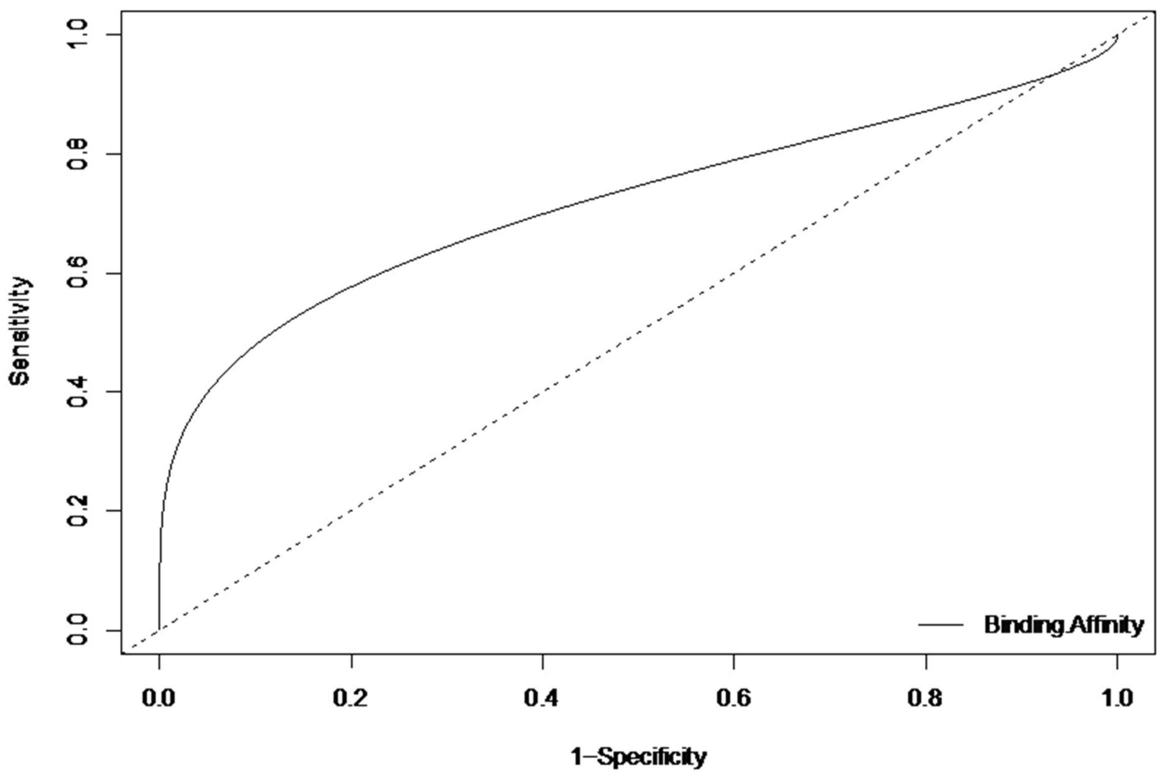

Figure S1. Receiver Operator Characteristic (ROC) curve generated after screening active molecules and their decoys against EBOV VP35 protein. The AUC value for the curve was 0.72, indicating the validity of the classification.

A)

86_complex

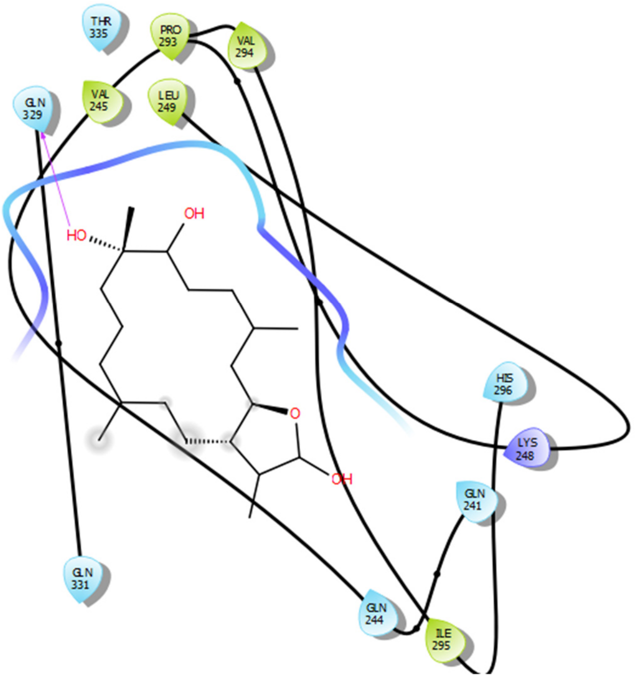

B)

95_complex

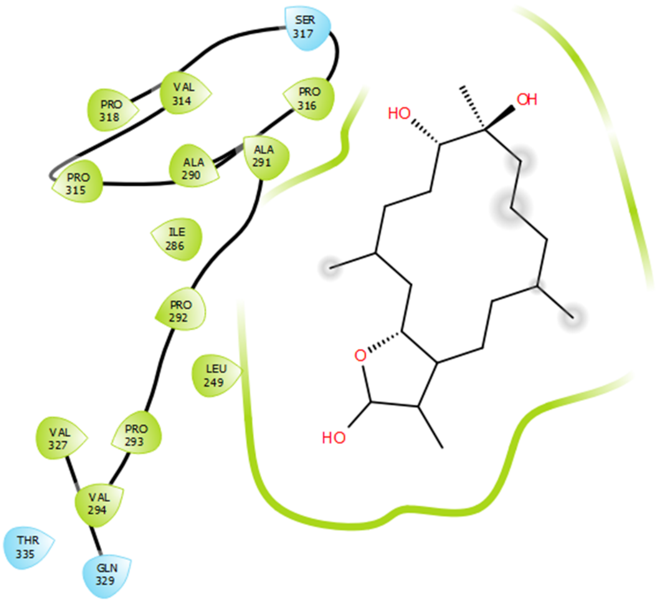

C)

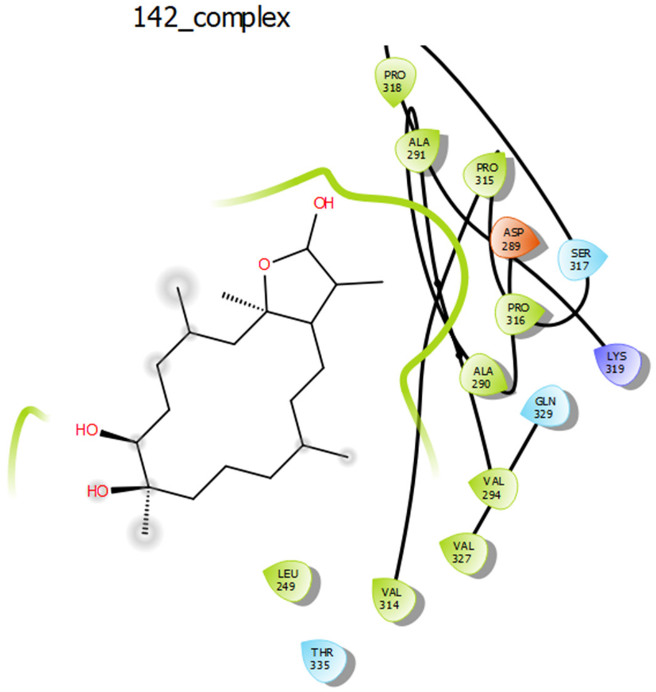
142_complex
PRO 318
ALA 291
PRO 315
ASP 289
SER 317
PRO 316
LYS 319
ALA 290
GLN 329
VAL 294
VAL 327
LEU 249
VAL 314
THR 335
OH
O
HO
HO

D)

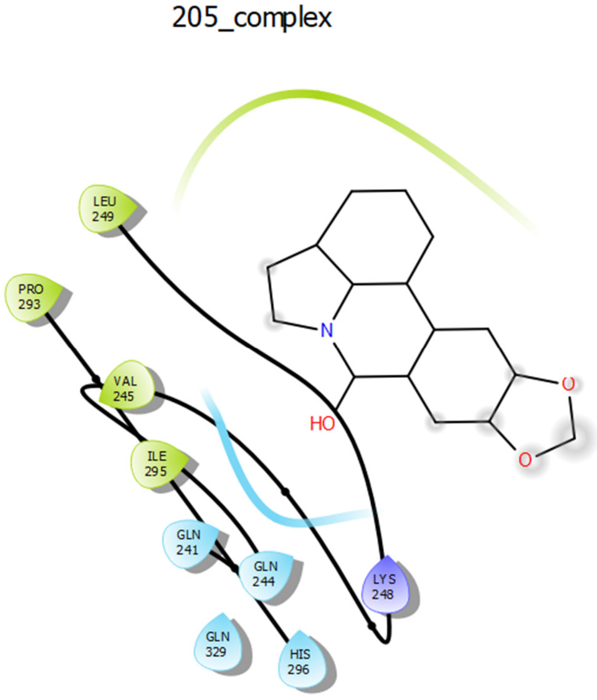
205_complex
LEU 249
PRO 293
VAL 245
ILE 295
GLN 241
GLN 244
GLN 329
HIS 296
LYS 248
N
HO
O
O

E)

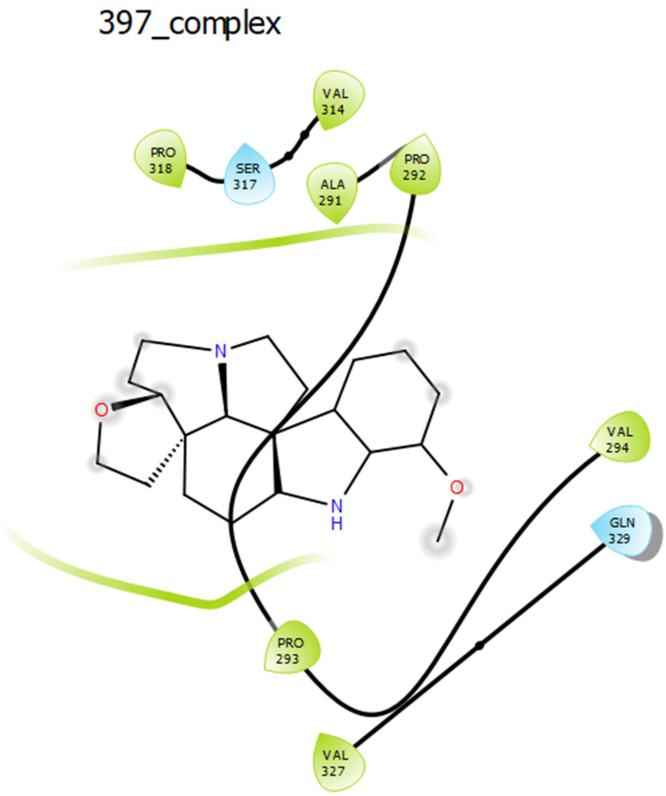
397_complex
VAL 314
PRO 318
SER 317
ALA 291
PRO 292
N
O
NH
O
VAL 294
GLN 329
PRO 293
VAL 327

F)

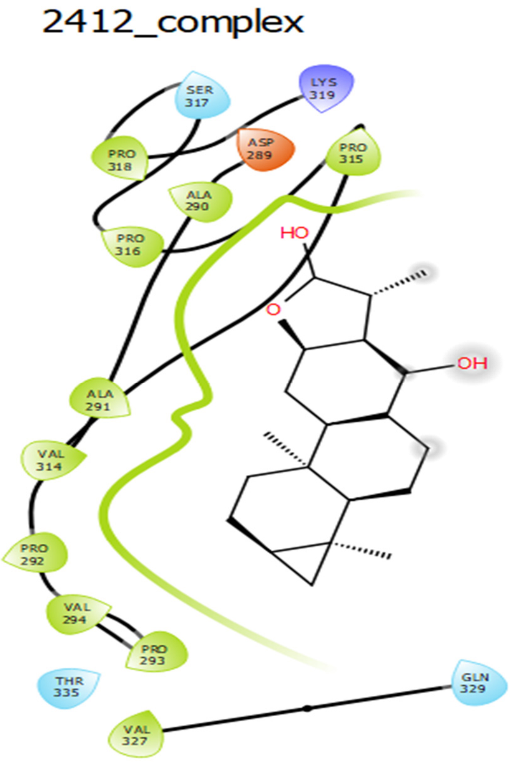
2412_complex
SER 317
LYS 319
PRO 318
ASP 289
PRO 315
ALA 290
PRO 316
HO
O
OH
ALA 291
VAL 314
PRO 292
VAL 294
PRO 293
THR 335
GLN 329
VAL 327

G)

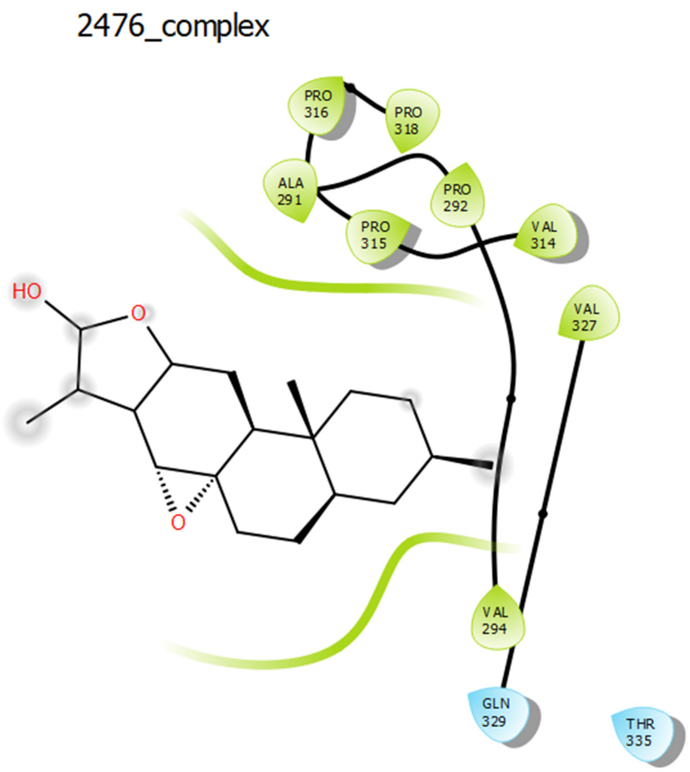
2476_complex
PRO 316
PRO 318
ALA 291
PRO 292
PRO 315
VAL 314
VAL 327
HO
O
O
VAL 294
GLN 329
THR 335

H)

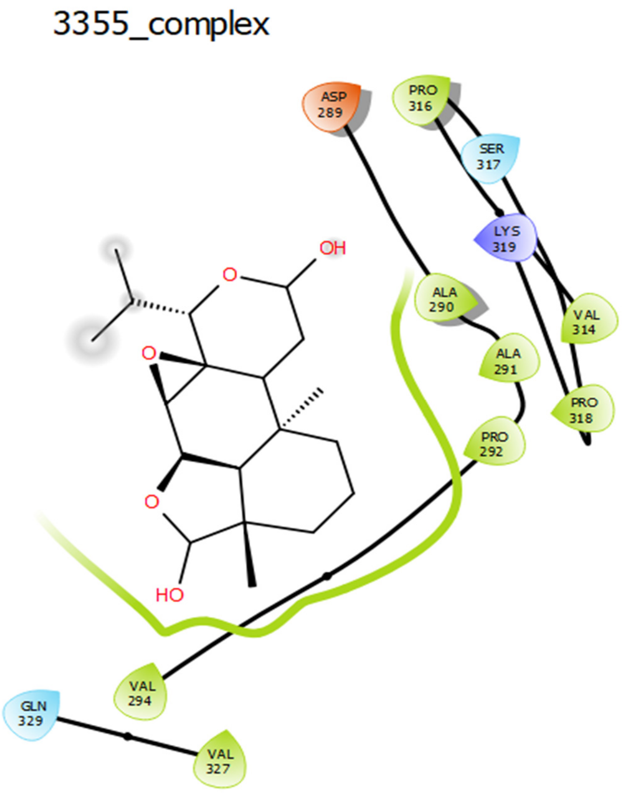
3355_complex
ASP 289
PRO 316
SER 317
LYS 319
OH
O
ALA 290
VAL 314
ALA 291
O
PRO 318
PRO 292
O
HO
VAL 294
GLN 329
VAL 327

I)

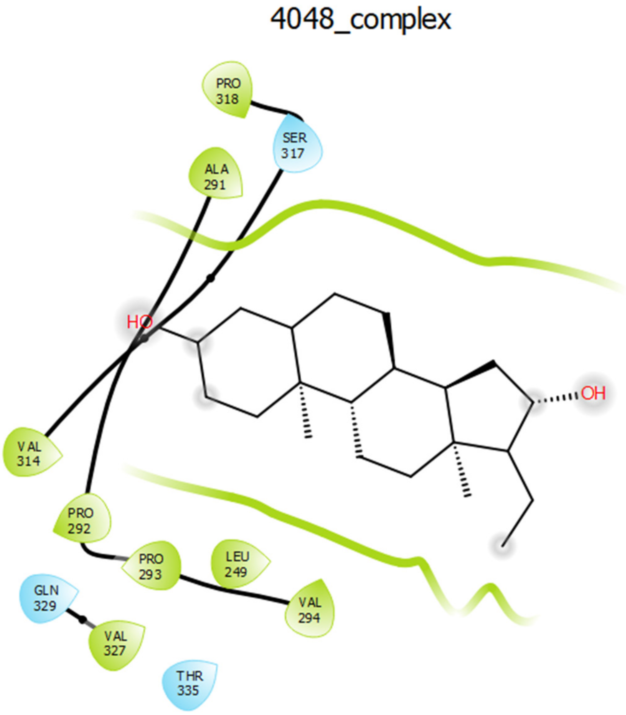
4048_complex
PRO 318
SER 317
ALA 291
HO
OH
VAL 314
PRO 292
PRO 293
LEU 249
VAL 294
GLN 329
VAL 327
THR 335

J)

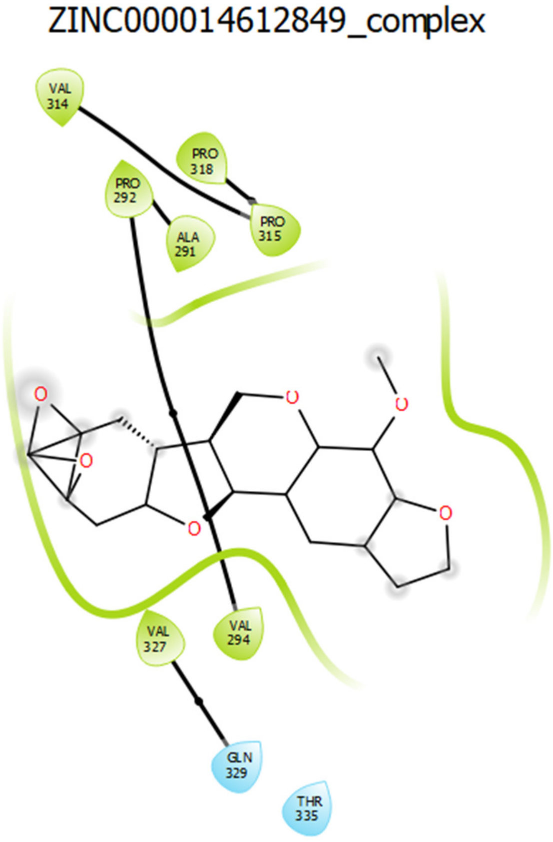
ZINC000014612849_complex
VAL 314
PRO 318
PRO 292
ALA 291
PRO 315
O
O
O
O
O
O
VAL 327
VAL 294
GLN 329
THR 335

K)

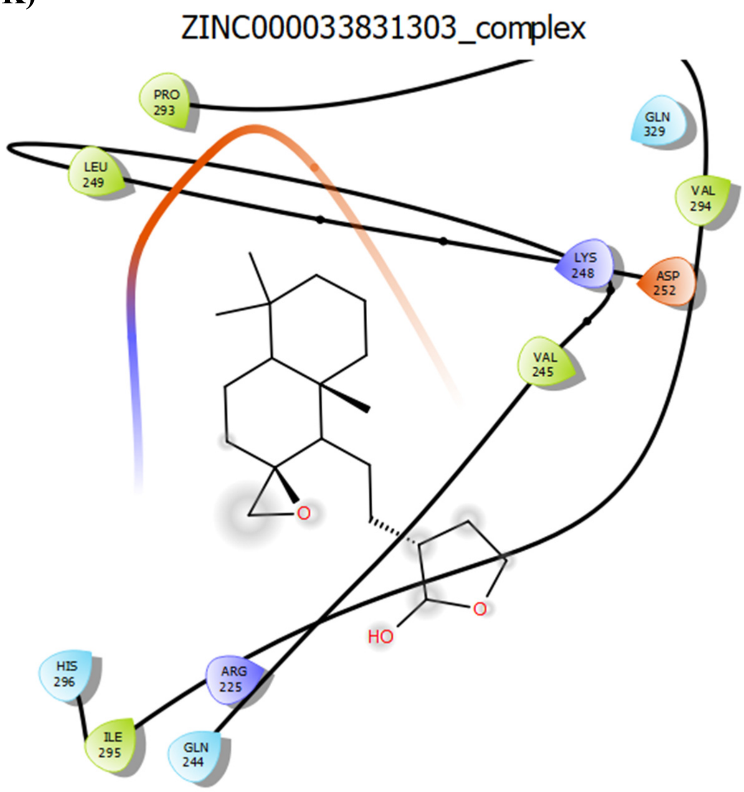

ZINC000033831303_complex
PRO 293
GLN 329
LEU 249
VAL 294
LYS 248
ASP 252
VAL 245
O
HO
O
HIS 296
ARG 225
ILE 295
GLN 244

L)

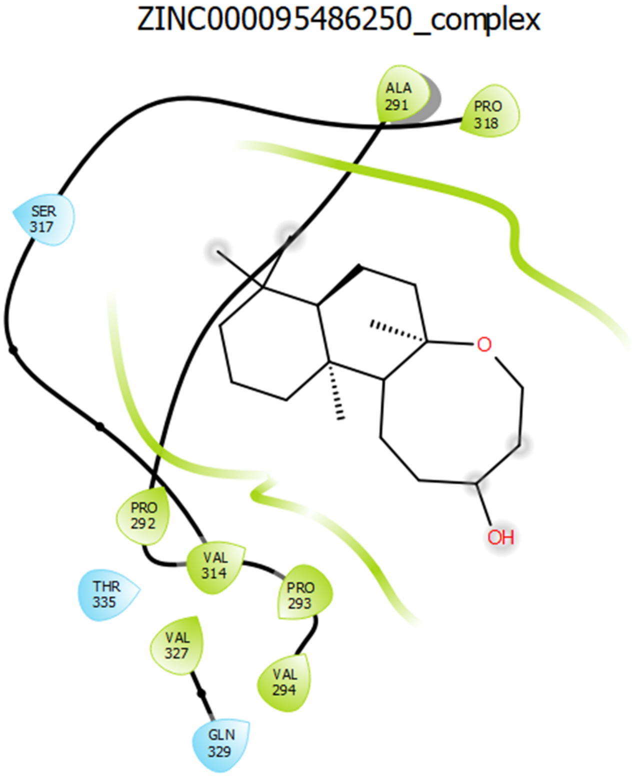

ZINC000095486250_complex
ALA 291
PRO 318
SER 317
O
OH
PRO 292
VAL 314
THR 335
PRO 293
VAL 327
VAL 294
GLN 329

M)

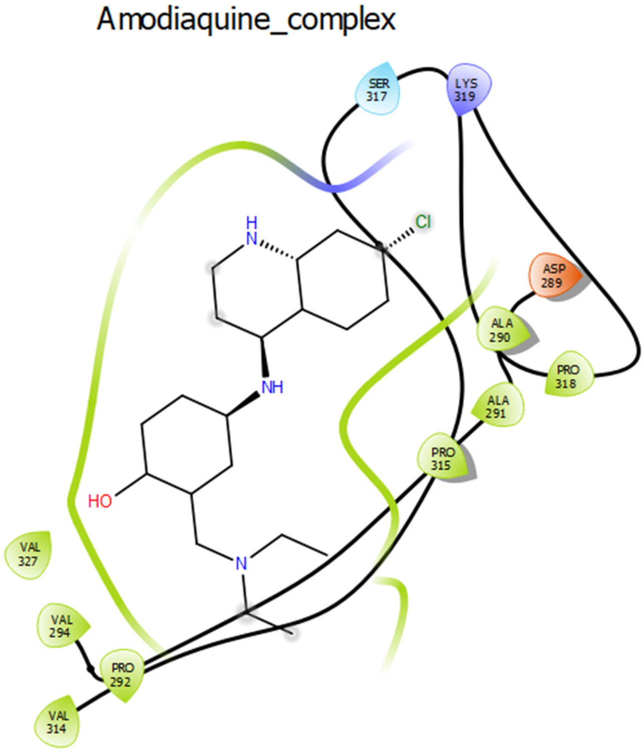
Amodiaquine_complex
SER 317
LYS 319
Cl
H N
ASP 289
ALA 290
PRO 318
NH
ALA 291
PRO 315
HO
N
VAL 327
VAL 294
PRO 292
VAL 314

N)

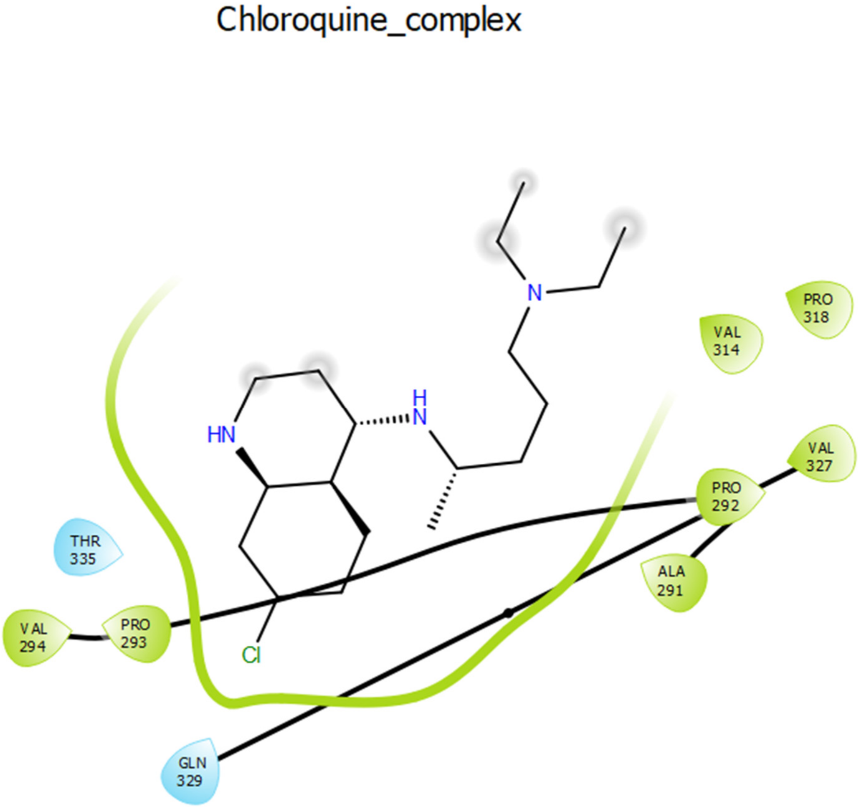
Chloroquine_complex
N
PRO 318
VAL 314
H N
HN
VAL 327
PRO 292
THR 335
ALA 291
VAL 294
PRO 293
Cl
GLN 329

O)

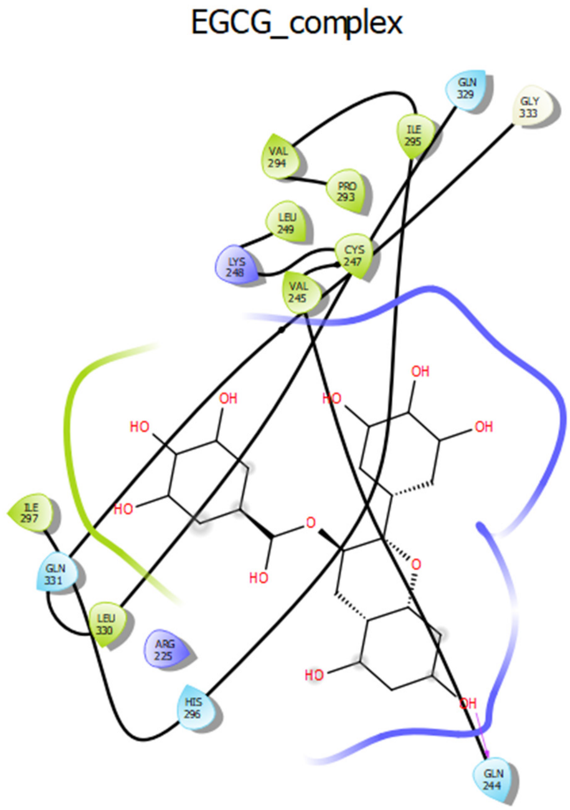
EGCG_complex
GLN 329
GLY 333
ILE 295
VAL 294
PRO 293
LEU 249
LYS 248
CYS 247
VAL 245
ILE 297
GLN 331
LEU 330
ARG 225
HIS 296
GLN 244

P)

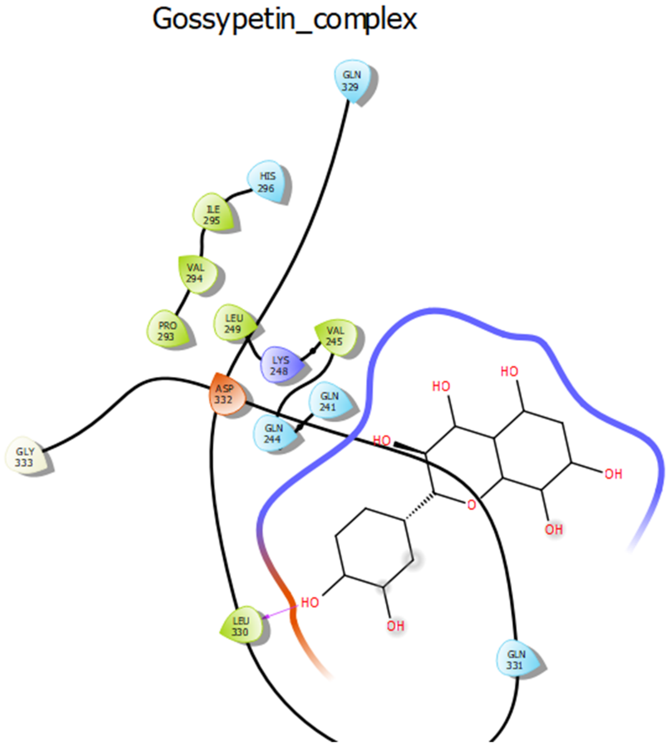
Gossypetin_complex
GLN 329
HIS 296
ILE 295
VAL 294
PRO 293
LEU 249
VAL 245
LYS 248
ASP 332
GLN 241
GLN 244
GLY 333
LEU 330
GLN 331

Q)

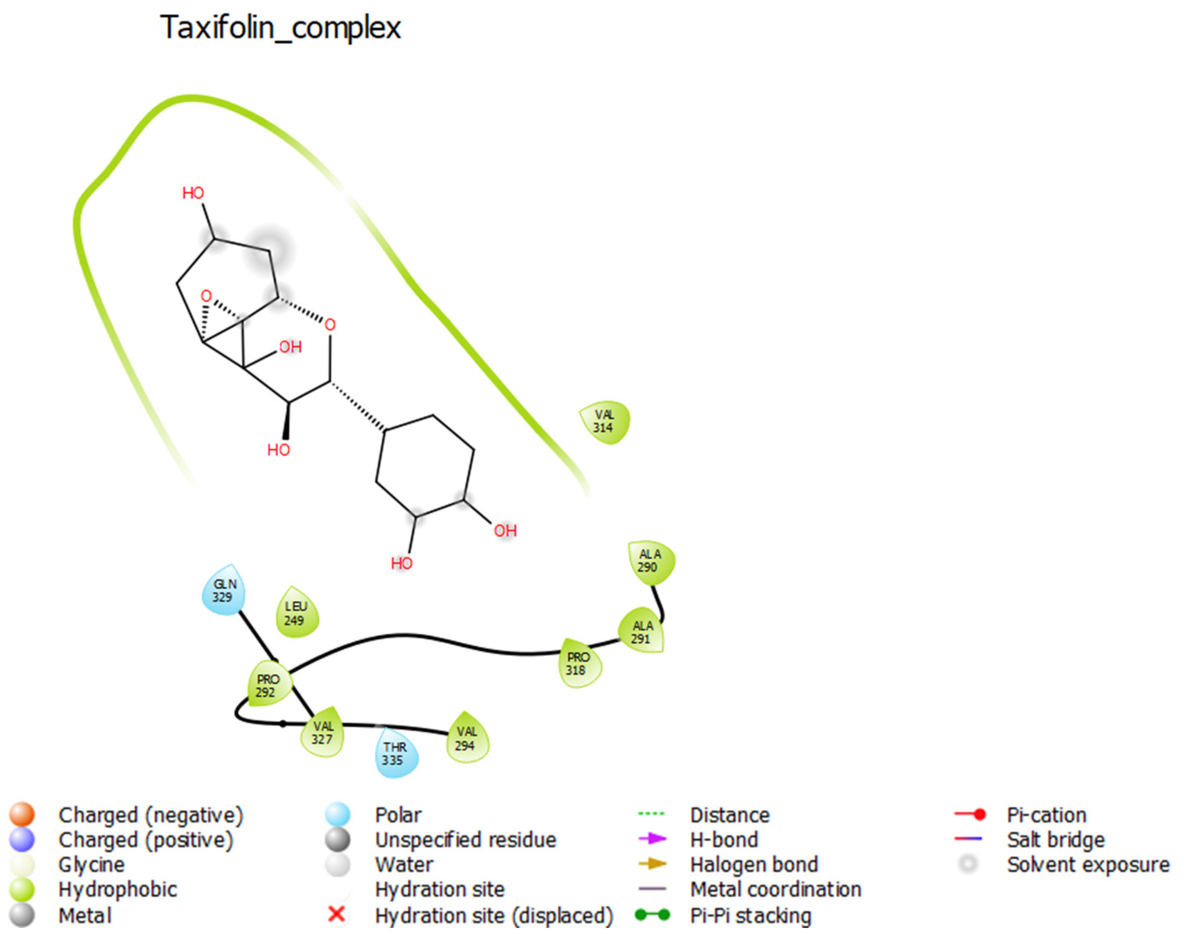

Figure S2. 2D diagrams of VP35-ligand complexes showing the hydrogen and hydrophobic interactions with the amino acid residues involved. The hydrogen bond with Gln329 is colored purple. The 2D protein-ligand interaction profile of EBOV VP35 in complex with (A) NANPDB86, (B) NANPDB95, (C) NANPDB142, (D) NANPDB205, (E) NANPDB397, (F) NANPDB2412, (G) NANPDB2476, (H) NANPDB3355, (I) NANPDB4048, (J) ZINC000014612849, (K) ZINC000033831303, (L) ZINC000095486250, (M) Amodiaquine, (N) Chloroquine, (O) EGCG, (P) Gossypetin, and (Q) Taxifolin.

Table S1. ADME prediction results of 12 compounds and 5 known inhibitors for Cytochrome P450 (CYP) inhibition.

Drug ID/Drug name	CYP1A2	CYP2C19	CYP2C9	CYP2D6	CYP3A4
NANPDB86	No	No	No	No	Yes
NANPDB95	No	No	No	No	No
NANPDB142	No	No	Yes	No	Yes
NANPDB205	No	No	No	No	No

NANPDB397	No	No	No	Yes	Yes
NANPDB2412	No	No	Yes	No	No
NANPDB2476	No	No	Yes	No	No
NANPDB3355	No	No	No	No	No
NANPDB4048	No	Yes	Yes	No	No
Amodiaquine	Yes	Yes	No	Yes	Yes
Chloroquine	Yes	No	No	Yes	Yes
EGCG	No	No	No	No	No
Gossypetin	Yes	No	No	Yes	Yes
Taxifolin	No	No	No	No	No

A)

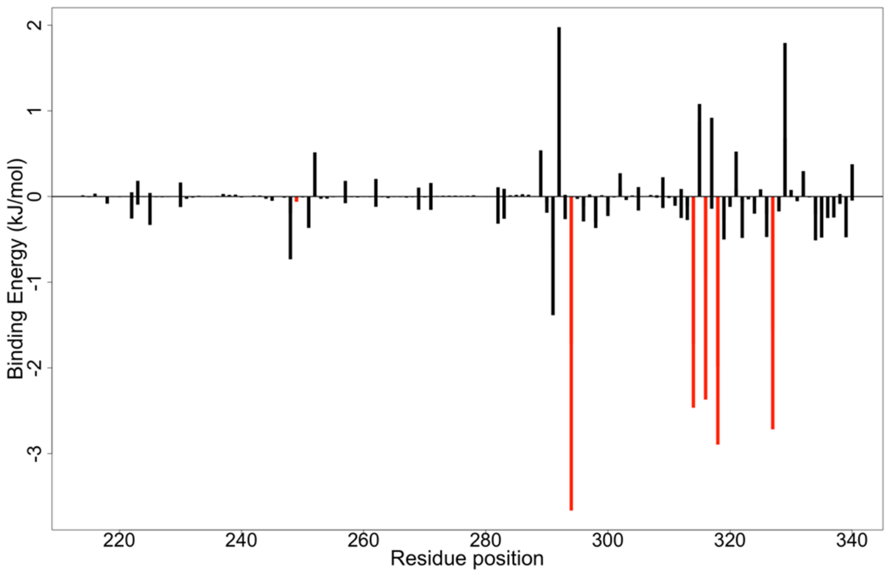

B)

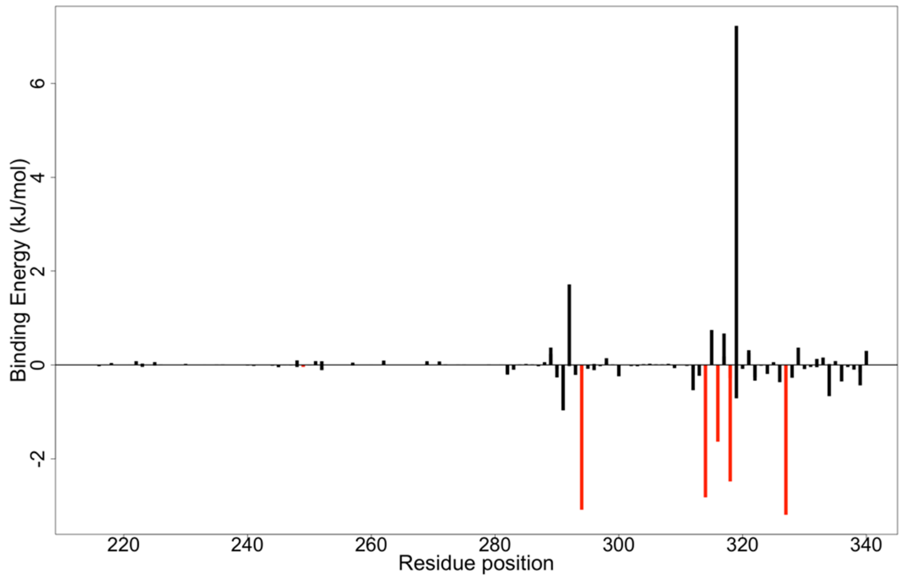

Binding Energy (kJ/mol)
-2
0
2
4
6
220
240
260
280
300
320
340
Residue position

C)

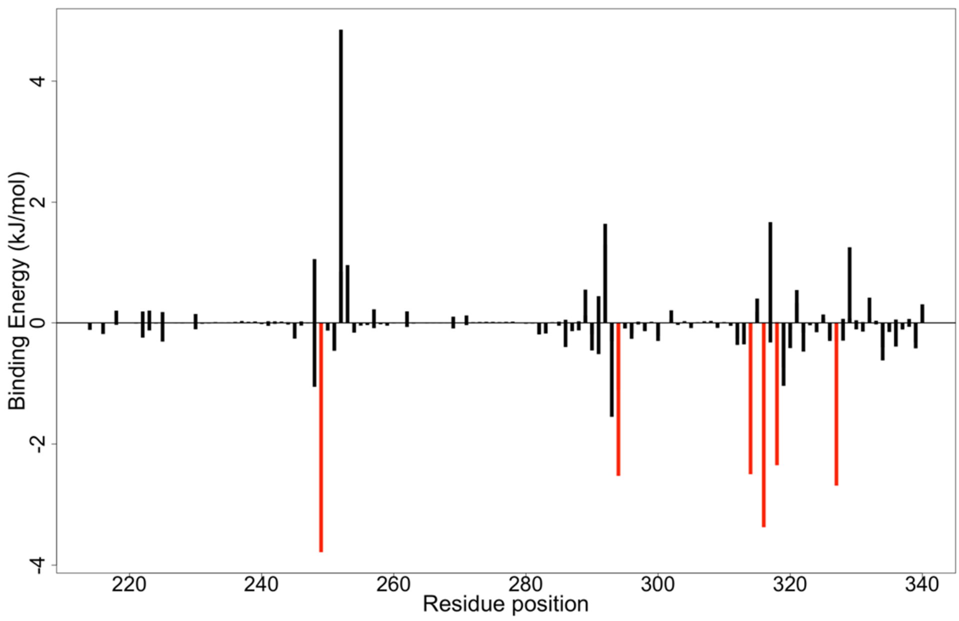

Binding Energy (kJ/mol)
-4
-2
0
2
4
220
240
260
280
300
320
340
Residue position

D)

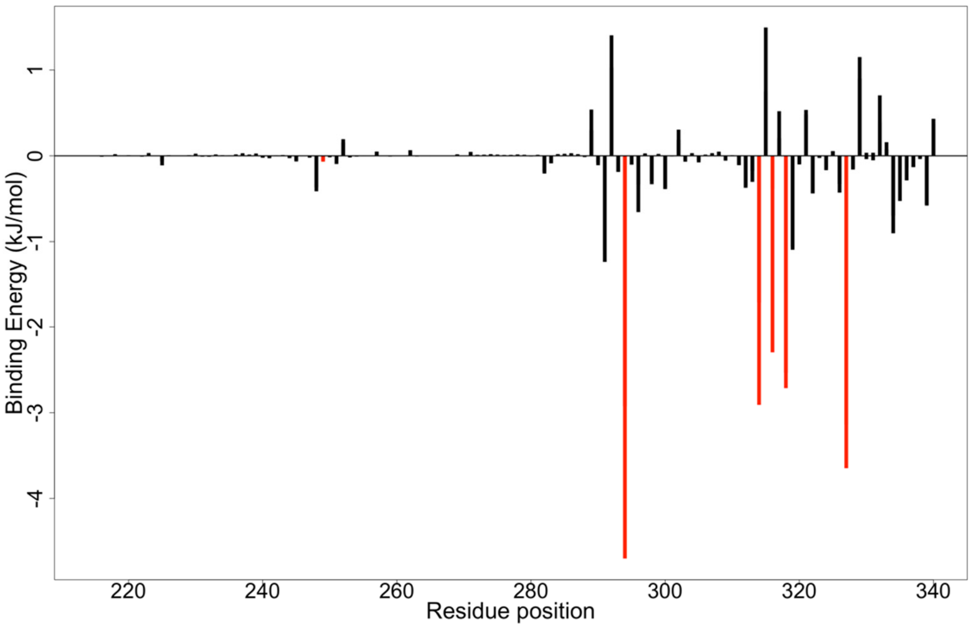

Binding Energy (kJ/mol)
1
0
-1
-2
-3
-4
220
240
260
280
300
320
340
Residue position

E)

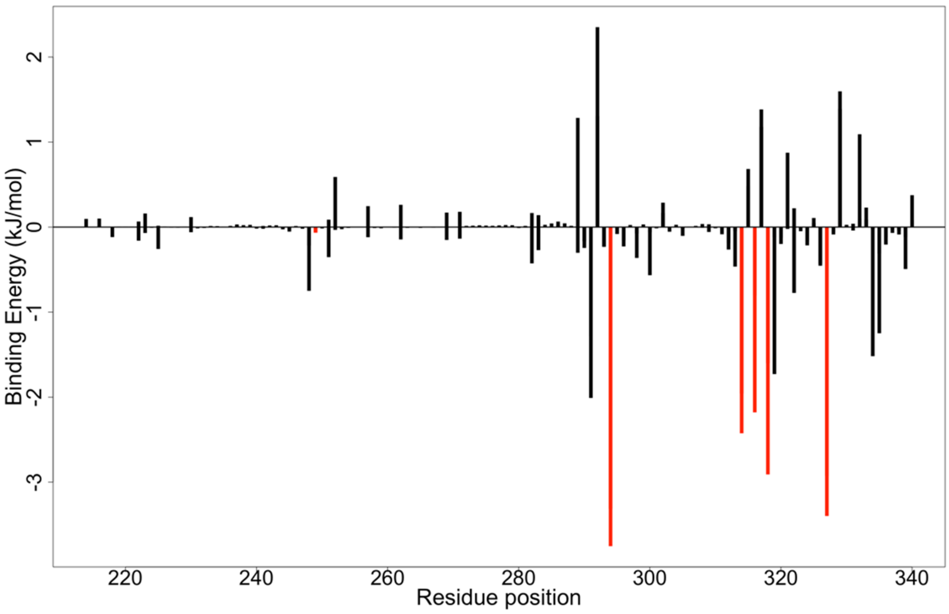

Binding Energy (kJ/mol)
2
1
0
-1
-2
-3
220
240
260
280
300
320
340
Residue position

F)

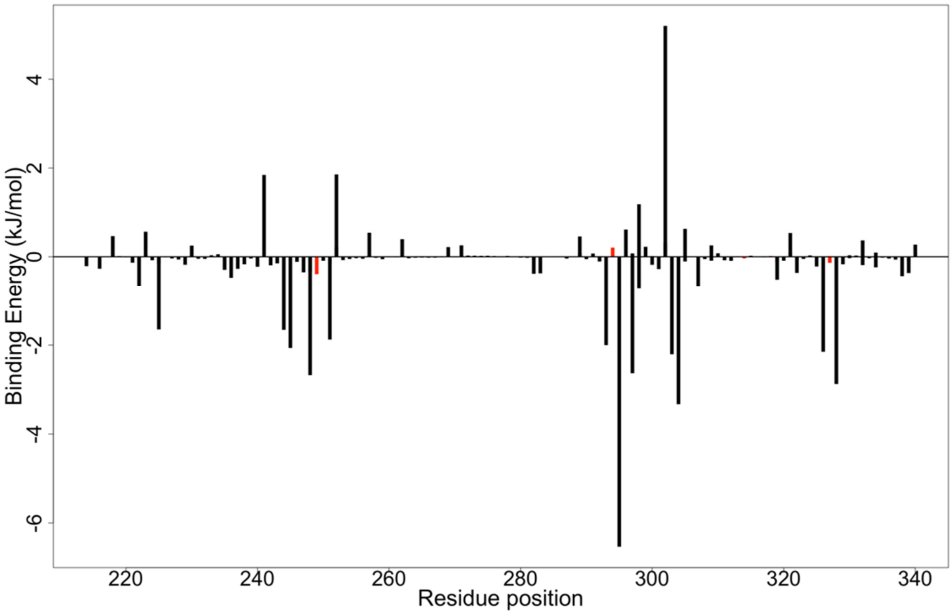

Figure S3. Molecular mechanics Poisson-Boltzmann surface area (MM-PBSA) plot of binding free energy contribution per residue of the VP35-ligand complexes: A) NANPDB2412, B) NANPDB2476, C) NANPDB4048, D) ZINC000095486250, E) Amodiaquine, and F) EGCG Fluctuations by predicted critical residues are shown in red.
